# Knockdown of stem cell regulator Oct4A in ovarian cancer reveals cellular reprogramming associated with key regulators of cytoskeleton-extracellular matrix remodelling

**DOI:** 10.1038/srep46312

**Published:** 2017-04-13

**Authors:** Chantel Samardzija, David W. Greening, Ruth Escalona, Maoshan Chen, Maree Bilandzic, Rodney Luwor, George Kannourakis, Jock K. Findlay, Nuzhat Ahmed

**Affiliations:** 1 Department of Obstetrics and Gynaecology, University of Melbourne, Victoria 3052, Australia; 2 Department of Biochemistry and Genetics, La Trobe Institute for Molecular Science, La Trobe University, Bundoora, Victoria 3086, Australia; 3 The Hudson Institute of Medical Research, Victoria 3168, Australia; 4 Fiona Elsey Cancer Research Institute, Ballarat, Victoria 3353, Australia; 5 Department of Surgery, University of Melbourne, Royal Melbourne Hospital, Victoria 3052, Australia; 6 Federation University Australia, Ballarat, Victoria 3010, Australia

## Abstract

Oct4A is a master regulator of self-renewal and pluripotency in embryonic stem cells. It is a well-established marker for cancer stem cell (CSC) in malignancies. Recently, using a loss of function studies, we have demonstrated key roles for Oct4A in tumor cell survival, metastasis and chemoresistance in *in vitro* and *in vivo* models of ovarian cancer. In an effort to understand the regulatory role of Oct4A in tumor biology, we employed the use of an ovarian cancer shRNA Oct4A knockdown cell line (HEY Oct4A KD) and a global mass spectrometry (MS)-based proteomic analysis to investigate novel biological targets of Oct4A in HEY samples (cell lysates, secretomes and mouse tumor xenografts). Based on significant differential expression, pathway and protein network analyses, and comprehensive literature search we identified key proteins involved with biologically relevant functions of Oct4A in tumor biology. Across all preparations of HEY Oct4A KD samples significant alterations in protein networks associated with cytoskeleton, extracellular matrix (ECM), proliferation, adhesion, metabolism, epithelial-mesenchymal transition (EMT), cancer stem cells (CSCs) and drug resistance was observed. This comprehensive proteomics study for the first time presents the Oct4A associated proteome and expands our understanding on the biological role of this stem cell regulator in carcinomas.

Ovarian cancer (OC) is the most lethal of all the gynaecological malignancies with a five-year mortality rate of >70%^1^. This poor outcome is due to the fact that the majority of OC cases are diagnosed at an advanced metastatic stage when the disease is no longer confined to the ovaries and is typically characterised by a widespread peritoneal dissemination and ascites^1^. While cytoreductive surgery and chemotherapy are initially effective in treating the disease in the short-term, relapse in advanced-stage patients is inevitable and almost all patients develop highly aggressive recurrent disease within few months which is intrinsically resistant to chemotherapy. Recent observations suggest OC recurrence may be driven by a sub-population of tumor cells which exhibit stem cell-like traits^2^,^3^. These cells, termed cancer stem cells (CSCs) not only display increased self-renewal characteristics as seen in embryonic stem cells (ESCs), but also exhibit tumorigenic survival properties and have been implicated in chemoresistance^4^,^5^. The molecular mechanisms which drive CSC-mediated OC progression, chemoresistance and recurrence have not yet been fully elucidated.

The presence and importance of CSCs in different cancer scenarios including OC has been accumulating for the last ten years. However, the origin and the biological identity of CSCs associated proteome still remains unclear. Several potential indirect mechanisms of CSC regulation have been proposed; of particular interest are the Notch, Hedgehog, Janus activated kinase/Signal transduction and activator of transcription (JAK/STAT), anti-apoptotic and drug-resistant pathways^5^,^6^,^7^. Others mechanisms include; malignant transformation of (i) adult stem cells into CSCs^8^,^9^; or (ii) multipotent progenitor or transit amplifying cells into CSCs^9^,^10^; or (iii) differentiated cells into CSCs which acquire stem cell characteristics after loss of differentiation ability^8^. A recent study has demonstrated the existence of equilibrium between CSCs and non-CSCs in a tumor with the balance being tipped towards CSCs in response to microenvironmental stimuli^11^,^12^. These candidate-based approaches though interesting does not elucidate the exact mechanism of CSC regulation which is absolutely essential to design CSC-based therapeutics required to abrogate clinically the residual tumor source which initiates recurrence.

Oct4 (Oct3/4, POU5F1) is a transcription factor which maintains self-renewal and pluripotency in embryonic stem cells and primordial germ cells^13^,^14^,^15^. The POU5F1 gene encodes two transcript variants, POU5F1A (Oct4A) and POU5F1B (Oct4B) which consist of 360 and 255 amino acids respectively, but share a common carboxyl-terminus of 225 amino acids^13^,^16^. Oct4B is generally localised in the cytoplasm, while Oct4A is localized mainly in the nucleus and has been associated with the maintenance of an undifferentiated state and stem cell properties of embryonic stem cells as well as primordial germ cells^15^,^16^. In addition, Oct4A expression has been shown as a diagnostic marker in germ cell tumors^17^. Recent studies have demonstrated elevated expression of Oct4 in several somatic tumors including breast, bladder, prostate, lung as well as of ovarian origin^13^. However, most studies have investigated Oct4 as a tumor marker; and only a handful of studies have reported expression analyses discriminating the Oct4A and Oct4B isoforms^13^,^18^. Hence, it remains undetermined whether Oct4 expression in most tumor groups is specific for stemness and/or CSCs, or it is just another tumorigenic marker used for expression analysis. Recently, transcriptomic, genomic and systems biology methods have identified Oct4 to be associated in an intricate regulatory network with Sox-2 and Nanog which results in the activation of transcription of genes required for pluripotency^19^,^20^. It is well established that the mRNA levels in a cellular system do not necessarily reflect protein abundance, and post-translational modification of proteins rapidly modulate protein activity and transduce signals crucial in maintaining stemness, differentiation, metastasis and drug resistance. However, the post-translational event of a cellular network which is important to map the regulatory mechanism of pluripotency or stemness cannot be identified by genomic and epigenomic studies and still remains obscured^21^. Hence elucidating the proteome of CSCs represents a rich informative repertoire of understanding cancer metastasis and recurrence.

MS-based proteomics has essentially changed the way by which malignant initiation and progression is investigated by its ability to identify and monitor thousands of proteins and post-translational modifications^22^. In OC, biomarker research for early-stage screening has become one of the most exciting uses for MS-based proteomics analysis^23^,^24^. Clinical specimens including tumors, ascites and serum samples have been assessed for distinct protein/peptide signatures to identify novel proteins which may assist in disease detection^25^. Recently, the methodology has also been used to identify novel therapeutic targets for recurrent diseases based on protein signatures of chemotherapy treated patients^26^. MS-based proteomics of CSCs therefore offers an advantage to study post-transcriptional regulation and signalling network of CSCs associated with self-renewal, differentiation, tumor progression as well as recurrence due to drug resistance. At the same time it provides a platform to study the proteome of a specific CSC marker in a targeted and high-throughput manner that allows dissection of crucial CSC-specific biology. It also provides avenues in dissecting fundamental differences between CSCs and adult stem cells, CSCs and embryonic stem cells and CSCs and pluripotent stem cells.

Using a large-scale, label-free quantitative MS-based proteomic profiling approach, this study for the first time identified novel proteins and/or peptides which are associated specifically with Oct4A in the HEY ovarian cancer cell line and an associated mouse xenograft model. By identifying specific protein targets and select protein networks associated with differential expression of Oct4A, this study aimed to contribute to our knowledge of the biological traits driven specifically by Oct4A in OC and potentially other tumor models.

## Methods and Materials

### Cell lines

The development of the HEY vector control and HEY Oct4A KD cell lines has been described previously^27^. SKOV3 and OVCAR5 cell lines have also been described previously^27^. Cells were grown in RPMI-1640 growth media supplemented with 10% (v/v) heat inactivated FBS (Thermo Fisher Scientific), 2 mM L-glutamine (Invitrogen, Australia) and an antibiotic combination of 1% (v/v) streptomycin and penicillin (Invitrogen, Australia). Cells were maintained at 37 °C in the presence of 5% CO_2_ and routinely checked for mycoplasma infection.

### Treatment of ovarian cancer cell lines with paclitaxel and cisplatin

Ovarian cancer cell lines were treated with paclitaxel and cisplatin at GI50 concentrations (50% growth inhibitory concentrations) for 72 hrs at 37 °C in the presence of 5% CO_2_ as described previously^27^. For paclitaxel treatment, HEY cells were treated with 1 ng/ml while SKOV3 and OVCAR5 cell lines were treated with 0.5 ng/ml. For cisplatin treatment of OVCAR5 cells a GI50 concentration of 3 μg/ml of cisplatin was used.

### Animal studies

Animal studies were carried out as described previously^27^.

### Whole cell lysates and tumor xenograft sample preparation

HEY vector control and HEY Oct4A KD cells were maintained in RPMI-1640 growth media as described previously^27^. After reaching >70% confluence, cells were washed three times in ice-cold PBS. Protein lysis buffer4% (w/v) SDS, 20% (v/v) glycerol and 0.01% (v/v) bromophenol blue, 0.125 M Tris-HCl, pH 6.8 containing complete EDTA-free protease inhibitor cocktail (Roche) and 1 mM Dithiothreitol was added directly to confluent cells and cells were then sonicated for 180 sec. Collected samples were incubated at 95 °C for 20 min and 60 °C for 2 hrs before being centrifuged at 25,000 × g for 30 min. The supernatant was stored at -80 °C until required.

For tumor xenograft samples, tumors were produced by intraperitoneal (i.p) injection of HEY Oct4A KD and HEY vector control cells into Balbc/c nude mice as previously described^27^. Sections of tumor xenografts were homogenised in lysis buffer and sonicated for 180 sec. Homogenates were then incubated at 95 °C for 20 min and 60 °C for 2 hrs before being centrifuged at 25,000 g for 30 min. Each supernatant sample was stored at -80 °C until required.

### Secretome Sample Preparation

Conditioned media (CM) collected from sub-confluent (80%) HEY vector control and HEY Oct4A KD cells grown in RPMI-1640 (serum-free) were centrifuged (500 × *g* for 5 min, 2000 × *g* for 10 min) CM concentrated by centrifugal ultrafiltration as described previously^28^. Each concentrated fraction was stored at -80 °C until required.

### Protein Quantification

The protein content of whole cell lysate and secreted cellular preparations was estimated by one-way dimensional SDS-PAGE/SYPRO Ruby protein staining densitometry as previously described^26^,^28^,^29^.

### Proteomic Analysis

Proteomic analyses were performed as previously described^29^ in biological replicates (n = 4) and technical duplicates (n = 2). Cell/tumor lysates and secreted sample preparations (10 μg protein) were lysed in SDS sample buffer(2% (w/v), 125 mM Tris-HCl, pH 6.8, 12.5% (v/v) glycerol, 0.02% (w/v) bromphenol blue), electrophoresed by short-range SDS-PAGE (10 × 6 mm), and visualized by Imperial Protein Stain (Invitrogen). Individual samples were excised, destained, reduced, alkylated, and trypsinized as described^26^. A nanoflow Ultra Performance Liquid Chromatography (UPLC) instrument (Ultimate 3000 RSLCnano, Thermo Fisher Scientific) was coupled on-line to a Linear Trap Quadropole (LTQ) Orbitrap Elite mass spectrometer (Thermo Fisher Scientific) with a nanoelectrospray ion source (Thermo Fisher Scientific). Peptides were loaded (Acclaim PepMap100, 5 mm × 300 μm i.d., μ-Precolumn packed with 5 μm C18 beads, Thermo Fisher Scientific) and separated (Acquity UPLC M-Class Peptide BEH130, C18, 1.7 μm, 75 μm × 250 mm, Waters). Data was acquired using Xcalibur software v2.1 (Thermo Fisher Scientific).

### Database searching and protein identification

Raw data were processed as described previously^29^ using Proteome Discoverer (v2.1, Thermo Fisher Scientific). MS2 spectra were searched with Mascot (v2.4, Matrix Science), and Sequest HT (v2.1, Thermo Fisher Scientific) against a database of 133,798 ORFs (UniProtHuman, Apr 2016). Peptide lists were generated from a tryptic digestion with up to two missed cleavages, carbamidomethylation of cysteines as fixed modifications, and oxidation of methionines and protein N-terminal acetylation as variable modifications. Precursor mass tolerance was 10 ppm, product ions were searched at 0.06 Da tolerances, minimum peptide length defined at 6, maximum peptide length 144, and max delta CN 0.05. Peptide spectral matches (PSM) were validated using Percolator based on q-values at a 1% false discovery rate (FDR). With Proteome Discoverer, peptide identifications were grouped into proteins according to the law of parsimony and filtered to 1% FDR. Scaffold Q + S (v4.5.3, Proteome Software Inc) was employed to validate MS/MS-based peptide and protein identifications from database searching. Initial peptide identifications were accepted if they could be established at greater than 95% probability as specified by the Peptide Prophet algorithm. Protein probabilities were assigned by the Protein Prophet algorithm. Protein identifications were accepted, if they reached greater than 99% probability and contained at least 2 identified unique peptides. These identification criteria typically established <1% false discovery rate based on a decoy database search strategy at the protein level. Proteins that contained similar peptides and could not be differentiated based on MS/MS analysis alone, were grouped to satisfy the principles of parsimony. Contaminants and reverse identification were excluded from further data analysis. UniProt was used for protein annotation.

### Label-free spectral counting, differentially expression and functional analysis

Significant spectral count (SpC) and Ratio of spectral count (Rsc) were determined as previously described^29^,^30^,^31^. The relative abundance of a protein within a sample was estimated using normalized SpC, where for each individual protein, significant peptide MS/MS spectra (i.e., ion score greater than identity score) were summated, and normalized by the total number of significant MS/MS spectra identified in the sample. The number of significant assigned spectra for each protein was used to determine protein differences between HEY Oct4A KD and the HEY vector control. For each protein the Fisher’s exact test was applied to significant assigned spectra. The resulting p-values were corrected for multiple testing using the Benjamini-Hochberg procedure^32^ and statistics performed as previously described^26^. Differentially expressed proteins were identified using the criteria: Rsc >±1.8 and p < 0.05.

### Protein-protein interaction analyses by STRING 10.0

Identification of entriched protein networks in cell lysates, secretomes and xenografts of Oct4AKD vs vector control was performed by STRING 10.0 software^33^. Clustering of proteins between samples was performed by Pearson correlation between samples using the protein profiles and visualized using gplots (https://cran.r-project.org/web/packages/gplots/index.html) package in R software. Raw data set of proteins identified in vector control and HEY KD samples (cell lysates, secretomes and xenografts) is described in Supplementary Table 1.

#### RNA extraction and Real-Time (RT) PCR

Quantitative real-time PCR was performed as described previously^27^. Relative quantification of gene expression was normalized to 18S and calibrated to the appropriate control sample using the SYBR Green-based comparative CT method (2^-ΔΔCt^). The primer set of Oct4A, vimentin (VIM), plectin (PLEC), TUBB2A and the house keeping gene 18S are described in Table 1.

#### Immunohistochemistry of mouse tumors

Immunohistochemistry analysis of mouse tumors was performed as described previously^4^,^5^,^27^. Briefly, formalin fixed, paraffin embedded 4 μm sections of the xenografts were dewaxed with Ventana EZ Prep and endogenous peroxidase activity was blocked using the Ventana’s Universal DAB inhibitor. Primary antibodies against Oct4, PLEC, VIM and TUBB2A were diluted according to the instruction provided by the manufacturer and sections were stained using a Ventana Benchmark Immunostainer (Ventana Medical Systems, Inc, Arizona, USA). Detection was performed using Ventana’s Ultra View DAB detection kit (Roche/Ventana, Arizona, USA) using the method described previously^4^. Tumor sections were counter stained with Ventana Haematoxylin and Blueing Solution. Immunohistochemistry images were captured and analysed by using Aperio ImageScope v12.1.0.5029 as described previously^27^.

## Results

### Proteome analysis of HEY vector control and Oct4A KD samples

**Figure 1:**
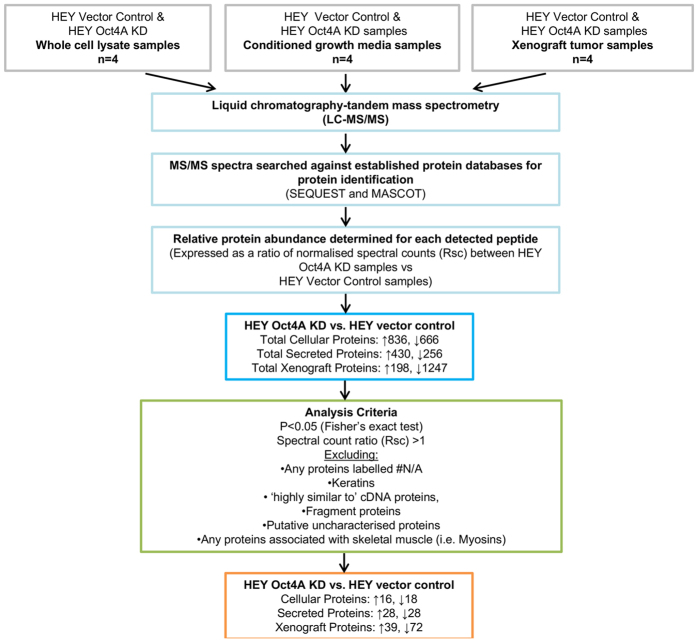
Schematic diagram of the methodology used to obtain protein lists from HEY Oct4A KD samples. HEY vector control and HEY Oct4A KD cells were prepared as whole cell lysate (n = 4), secretome (n = 4) and xenograft tumor samples (n = 4). Samples were solubilised, separated by short-range SDS-PAGE and subjected to in-gel reduction, alkylation, and tryptic digestion. Extracted peptides were fractionated and identified using mass spectrometry analysis, data processing database searching, informatics and protein annotation. Relative protein abundance was determined by estimating the ratio of normalised spectral counts (Rsc) between HEY Oct4A KD samples and HEY vector control samples for each protein. To determine the classification of proteins in response to Oct4A we applied a stringent analysis filtering criteria. The number of proteins which met the selection criteria for each HEY Oct4A KD sample group is listed.

We have recently shown knockdown of Oct4A in a HEY cell line by small hairpin (sh)RNA technology^27^,^34^. Knockdown of Oct4A in the HEY cell line (Oct4AKD) was confirmed at the protein level by Western blot and immunofluorescence and at the mRNA level by Quantitative Real Time Polymerase Chain Reaction (RT-PCR)^27^,^34^. MS-based proteomics analysis on vector control and Oct4A KD cells showed a total of 836 proteins to be up regulated in HEY Oct4A KD cellular samples compared to HEY vector control samples, while 666 cellular proteins were down regulated in HEY Oct4A KD samples (biological n = 4, technical duplicate) (Fig. 1). For secreted proteins, a total of 430 proteins were up-regulated in HEY Oct4A KD samples compared to HEY vector control, while 256 proteins were down regulated. In mouse xenograft tumors, 198 proteins were up regulated in HEY Oct4A KD samples compared to HEY vector control samples, while 1247 proteins were down-regulated (Fig. 1).

### Protein selection criteria

Following data collection and bioinformatics analyses, proteins which were not differentially expressed (p < 0.05) in HEY Oct4A KD samples when compared to HEY vector control samples were eliminated. Proteins identified as keratins were also removed from analysis based on known contaminants involved in proteomics analysis^35^. Proteins which had no known protein accession number were also excluded based on the fact that they could not be identified. Other exclusion criteria included any protein identified as a peptide fragment, a putative uncharacterised protein or those which are cDNA-like in nature. This was based on the fact that an accurate protein function may not be identified for these specific peptides. Proteins detected in tumor xenograft samples which were related to skeletal muscle were also excluded from the study based on likelihood of skeletal muscle contamination during tumor xenograft excision. An outline of the protein selection process is described in Fig. 1.

**Figure 2:**
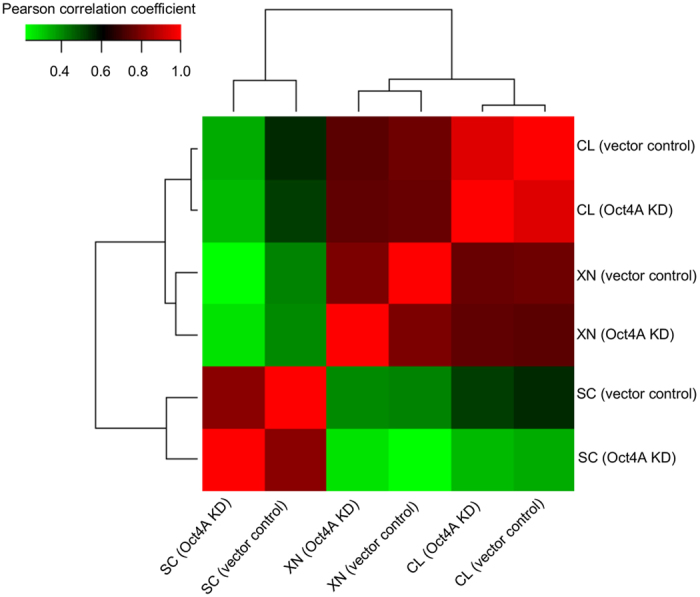
Characterisation of HEY Oct4A subsets reveal correlation between sample subsets in response to Oct4A expression. Correlation matrix of cell lysates (CL), tumor xenografts (XN), and secretomes (SC) samples representing differential abundance based on normalised spectral count (SpC) values between HEY Oct4A KD and HEY vector control samples. Correlation expression profile reveals that each individual sample represents clear distribution and similarity with other sample subsets.

A correlation plot between the samples is presented in Fig. 2. This cluster and normalised heat map analysis revealed that there was a high correlation between different samples (i.e., cell lysates, xenograft lysates and secreted). HEY vector control and Oct4A KD cell samples were most similar in expression profiles, while similarities were identified in cell and xenograft lysates and secreted samples. These protein expression cluster analyses highlight key differences in protein expression between HEY vector control and Oct4A KD sample subsets.

### Proposed protein functions of differentially expressed HEY Oct4A KD cellular proteins

#### Down regulated cellular proteins in HEY Oct4A KD cells compared to Oct4A vector control cells

**Figure 3:**
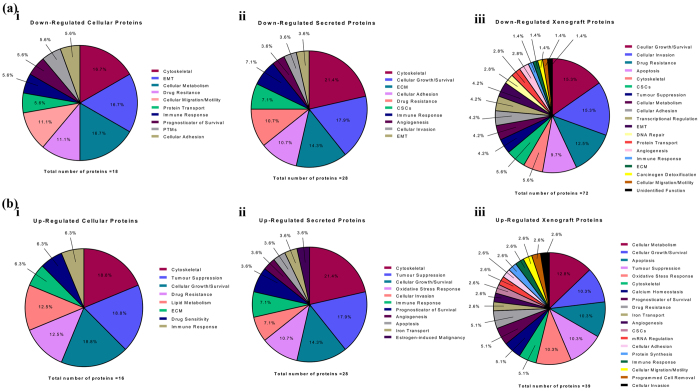
Distribution of the potential biological functions of Oct4A protein targets in HEY Oct4A KD cells. The functions of the Oct4A protein targets obtained from the HEY Oct4A KD cells were searched in the literature and categorised according to their potential cancer-related biological functions. **(a)** Differentially expressed down regulated proteins are summarised as: (i) down regulated cellular proteins, (ii) down regulated secreted proteins, (iii) downregulated tumor xenograft proteins; **(b)** Differentially expressed up-regulated proteins are summarised as: (i) up-regulated cellular proteins, (ii) up-regulated secreted proteins, (iii) up-regulated tumor xenograft proteins.

From the selection criteria stipulated in Fig. 1, a total of 18 cellular proteins were identified to be differentially down regulated in HEY Oct4A KD samples when compared HEY vector control samples (Fig. 1). When classified according to their cellular function, proteins which fit into the categories of cytoskeletal regulation, EMT and cellular metabolism constituted 16.7% and were identified as the most frequently down-regulated proteins in HEY Oct4A KD cellular samples (Table 2, Fig. 3a). This was followed by proteins which were related to drug resistance and cellular migration/motility which constituted 11.1% of each category, followed by protein transport, immune response, prognosticator of survival, post-translational modifications, and cellular adhesion which constituted 5.6% of each category of down regulated proteins (Table 2, Fig. 3a). Of all the identified down regulated cellular proteins, the cytoskeleton-related Tubulin beta-2A chain protein (TUBB2A) was the most abundantly down regulated (Rsc -42.9), followed by the EMT-related protein 14-3-3ε (YWHAE) (Rsc −10.0). Other down regulated cellular proteins which were identified to be of interest included the cellular migration and invasion related actin-binding protein Swiprosin-1 (EFHD2) (Rsc −4.1), the drug resistance-related proteins Glyoxalase 1 (GLO1) (Rsc −3.1) and actin-binding protein Trangelin-2 (TAGLN2) (Rsc −3.0) cellular metabolism-related protein Enoyl-CoA Hydratase 1 (ECHS1) (Rsc −3.7) and cytosketelal as well as extracellular remodelling proteins PLEC (Rsc −1.1) and VIM (Rsc −1.2). A full list of down-regulated cellular proteins and their proposed cancer-related classifications are listed in Table 2 and summarised in Fig. 3a.

#### Up regulated cellular proteins in HEY Oct4A KD samples

Compared to HEY vector control samples, a total of 16 differentially up regulated cellular proteins were identified in HEY Oct4A KD samples (Fig. 1). Proteins which are categorised to be associated with cytoskeleton, tumor suppression, cellular growth, as well as those involved in lipid metabolism and drug resistance were identified as the most frequently up regulated in HEY Oct4A KD cellular samples (Fig. 3b, Table 3). The most significantly differential expressed cellular protein identified in HEY Oct4A KD samples was the cytoskeleton-related actin binding protein Plastin-1 (PLS1) (Rsc 7.1). Other up regulated cellular proteins which were identified included the cytoskeletal-related actin binding protein Twinfilin-1 (TWF1) (Rsc 5.1), and the tumor suppression-related proteins Apolipoprotein A-1 (APOA1) (Rsc 5.1) and Eukaryotic release factor 1 (ETF1) (Rsc 2.3). A list of up regulated cellular proteins and their proposed cancer-related classifications are described in Table 3 and summarised in Fig. 3b.

### Proposed protein functions of differentially expressed HEY Oct4A KD secreted proteins

#### Down regulated secreted proteins in HEY Oct4A KD samples

Analysis of the secretome revealed a total of 28 proteins were differentially suppressed in HEY Oct4A KD samples compared to HEY vector control samples (Fig. 1). When classified according to their function, a large number of proteins were found to be involved in cytoskeletal functions and cellular growth (Table 4). This was followed by proteins known to be involved with the ECM, CSCs, cellular adhesion and drug resistance. Of the identified down regulated secreted proteins, the ECM-related protein Fibronectin 1 (FN1) was the most significantly differentially expressed protein in HEY Oct4A KD samples (Rsc −15.3). This was closely followed by the ECM-related protein Laminin subunit gamma-2 (LAMC2) (Rsc −10.2). Other down regulated secreted proteins which were identified to be of interest included the ECM-related protein Laminin subunit beta-3 (LAMB3) (Rsc −7.8), the cytoskeleton cellular invasion-associated protein PLEC (Rsc −6.7), the CSC-associated protein CD109 antigen (CD109) (Rsc −5.0) and the drug resistance-related protein and protein identified as concurrently down-regulated in HEY Oct4A KD cellular samples Transgelin-2 (TAGLN2) (Rsc −4.7). Secreted proteins suppressed in HEY Oct4A KD samples and their proposed cancer-related functions are listed in Table 4 and summarised in Fig. 3a.

#### Up regulated secreted proteins in HEY Oct4A KD samples

Twenty-eight secreted proteins were differentially over-expressed in HEY Oct4A KD conditioned media samples when compared to HEY vector control samples (Fig. 1). Secreted proteins which were identified to have functional roles in cytoskeletal regulation, tumor suppression and cellular growth were found to be the most significantly up regulated in HEY Oct4A KD samples (Table 5). This was followed by proteins involved in oxidative stress response, inflammation and proteins used as prognosticators of survival. The most abundantly up regulated protein was the tumor suppressor-related protein HBB (Rsc 5.0). This was closely followed by the estrogen induced malignancy-associated protein Thyroxine-binding globulin (SERPINA7) (Rsc 4.7). Other up-regulated secreted proteins of interest included the immune response-associated protein Pentraxin-related protein 3 (PTX3) (Rsc 4.1), the oxidative stress response-related protein Glutathione S-transferase phosphate 1 (GSTP1) (Rsc 3.3) and the apoptosis-related protein POTE ankyrin domain family member F (POTEF) (Rsc 2.6). Similar to that identified in HEY Oct4A KD cellular samples, the tumor suppression-associated protein Apolipoprotein A1 (APOA1) was also up regulated in conditioned media preparations of HEY Oct4A KD samples compared to HEY vector control samples (Rsc 4.3). Up regulated secreted proteins and their proposed cancer-related classifications are listed in Table 5 and summarised in Fig. 3b.

### Proposed protein functions of differentially expressed HEY Oct4A KD xenograft tumor proteins

#### Down regulated xenograft tumor proteins in HEY Oct4A KD samples

A total of 72 proteins were differentially suppressed in xenograft tumors derived from HEY Oct4A KD cells when compared to xenograft tumour samples derived from HEY vector control cells (Fig. 1). The increased number of proteins identified to be decreased in tumor xenograft samples resulted in an extensive list of protein function categories. Proteins which fit into the categories of cellular growth, cellular invasion, drug resistance, apoptosis, cytoskeletal and CSCs were identified as the most frequently down regulated proteins in HEY Oct4A KD tumor xenograft samples. This was followed by proteins which were involved in cellular adhesion, EMT, cellular metabolism and tumor suppression. The most down regulated protein identified in HEY Oct4A KD xenograft tumor samples was the cytoskeleton-associated protein TUBB2A (Rsc −78.1). This was followed by the cellular adhesion-associated proteins FN1 (Rsc −57.2) and FN cleaved into Anastellin (Rsc −55.4). Other down regulated proteins of interest in tumor xenografts derived from HEY Oct4A KD cells included the CSC-associated proteins CD109 antigen (CD109) (Rsc −12.8) and Pyruvate dehydrogenase E1 component subunit beta (PDHB) (Rsc −11.9), the EMT-related proteins Transforming growth factor-beta-induced protein (TGFβ1) (Rsc −12.8) and Plastin-3 (PLS3) (Rsc −4.7), the cellular adhesion-associated proteins integrin-linked protein kinase (ILK) (Rsc −5.6) and intergin alpha-2 (ITGA2) (Rsc −2.9), the cellular migration and invasion-associated proteins PLEC (Rsc −3.2), (VIM) (Rsc −2.3) and Annexin 6 (ANXA6) (Rsc −2.9) and the angiogenesis-related protein Thioredoxin Reductase 1 (TXNRD1) (Rsc −4.7). Down regulated xenograft related proteins and their proposed cancer-related classifications are listed in Table 6 and summarised in Fig. 3b.

#### Up regulated xenograft tumor proteins in HEY Oct4A KD samples

Thirty-nine proteins were identified to be differentially elevated in tumor xenografts derived from HEY Oct4A KD cells compared to tumors derived from HEY vector control cells. Xenograft proteins which were identified to have functional roles in cellular growth, cellular metabolism, apoptosis, tumor suppression and oxidative stress response were the most frequently up regulated in HEY Oct4A KD tumor xenografts. This was followed by proteins involved with the cytoskeleton, calcium homeostasis and drug resistance. The most up regulated protein identified in HEY Oct4A KD xenograft tumor samples was the apoptosis-associated protein POTEF (Rsc 23.0). Other up regulated tumor xenograft proteins identified to be of interest included the tumor suppression-related protein Alpha amylase (AMY2A) (Rsc 7.3), the cellular growth-associated protein Eukaryotic translation initiation factor 4A2 (EIF42A) (Rsc 5.9), the drug resistance-associated protein Collagen alpha 3 (VI) chain (COLGA3) (Rsc 3.7) and the cellular metabolism-related protein Isocitrate dehydrogenase (IDH2) (Rsc 2.5). Up regulated xenograft tumor proteins and their proposed cancer-related classifications are listed in Table 7 and summarised in Fig. 3b.

#### Proteome data network and pathway analysis

**Figure 4:**
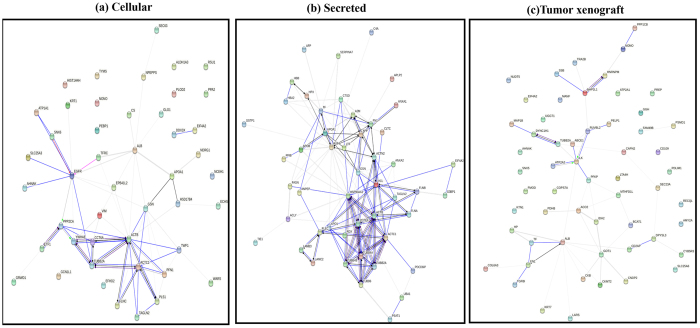
Protein interaction network analysis of secreted differentially expressed proteins. Protein interaction network generated with STRING 10.0 for, (**a**) cellular, (**b**) secretome and (**c**) tumor xenograft and samples. Based on molecular high-confidence action and functional enrichments analysis, major clusters of interacting proteins include those involved in regulation of actin cytoskeleton, focal adhesion, and tubulin (secretome), focal adhesion, adherenes junctions, cytoskeleton, extracellular region, and cell junction protein networks (cellular), and metabolic processes (carboxylic acid, oxoacid, carbohydrate), extracellular region, and cytoskeleton (tumor xenograft).

To identify protein networks and clusters associated with differentially expressed proteome profiles from HEY vector control and Oct4A KD cellular (41 significantly differentially expressed proteins), secretome (59 significantly differentially expressed proteins) and xenograft (57 significantly differentially expressed proteins), we performed protein-protein interaction analyses by STRING 10.0^33^ (Fig. 4a–c). Several clusters of interacting proteins in vector control compared to Oct4A KD cells were observed, including focal adhesion, adheren junctions, cytoskeleton, extracellular region, and cell junction protein networks (Fig. 4a), while for the secretome several clusters of interacting proteins in vector control compared to Oct4A KD cells included regulation of actin cytoskeleton, focal adhesion, and tubulin protein networks (Fig. 4b). On the other hand, tumor xenograft samples included clusters of interacting proteins regulating metabolic processes (carboxylic acid, oxoacid, and carbohydrate), extracellular region, and cytoskeleton protein networks (Fig. 4c).

#### Validation of candidate proteins by RT-PCR and immunohistochemistry

**Figure 5:**
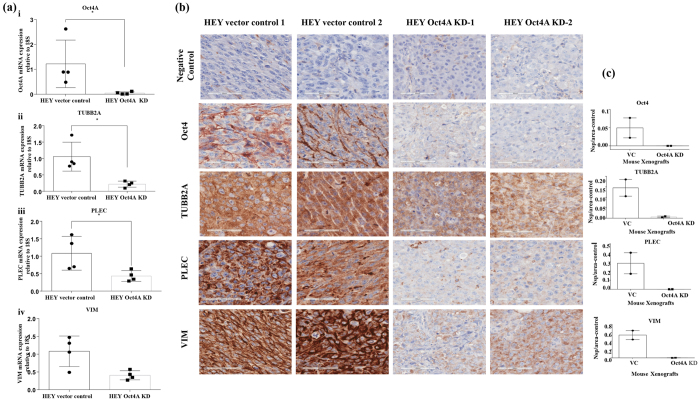
(**a**) Expression of Oct4A, TUBB2A, PLEC and VIM in HEY vector control and Oct4A KD cells. RNA from HEY vector control and HEY Oct4A KD cells was extracted, cDNA was prepared and RT-PCR was performed as described in the Materials and Methods. The resultant mRNA levels were normalized to 18S mRNA. Results are representation of three independent experiments performed in triplicate. Significant variations between vector control and Oct4A KD cells was analysed by unpaired t-test with Welch’s correction using GraphPad Prism 7.02 and are indicated by *P < 0.05. **(b**) Expression of Oct4, TUBB2A, PLEC and VIM in mouse tumor xenografts generated by HEY vector control and Oct4A KD cells. Representative immunohistochemistry images of mouse xenografts for the expression of Oct4A, TUBB2A, PLEC and VIM. Images are set at 400x magnification and scale bar represents 60 μM **(c)** Variations in staining were determined by subtracting the negative control DAB readings number of strong positivity (Nsp)/area from the DAB readings of protein of interest for each xenograft. Data is presented as the mean ± SEM.

To validate the expression of selected proteins from proteomic profiling between the HEY Oct4A vector control and HEY Oct4A KD populations, RT-PCR and immunohistochemistry were carried out on a subset of proteins (TUBB2A, PLEC, VIM) in Oct4A vector control and Oct4A KD cells and associated xenografts (Fig. 5a and b). The three proteins selected for validation were significantly down regulated in HEY Oct4A KD cells and KD-derived xenografts compared to vector control cells and associated xenografts (Suppl Table 1). These proteins were chosen as they have known significant role in cytoskeletal and ECM reprogramming of cancer cells (Suppl File 1).

For RT-PCR analysis, the expression of Oct4A was significantly greater in HEY vector control compared to HEY KD cells (Fig. 5a). This elevated expression of Oct4A was consistent with the immunohistochemistry staining of det4in HEY Oct4A vector control compared to HEY Oct4A KD cell-derived xenografts (Fig. 5b). Consistent with that, RT-PCR analysis also showed consistent down regulation of TUBB2A, PLEC and VIM in HEY Oct4A KD compared to Oct4A vector control cell lysates (Fig. 5a). The down regulation of TUBB2A, PLEC and VIM in KD cell lysates was coherently observed in Oct4A KD cells-derived xenografts compared to vector control cell-derived xenografts. These validations of the differential expression of Oct4A, TUBB2A, PLEC and VIM at the mRNA and protein levels in cell lysates and the xenografts is in harmony with the comprehensive proteomics data demonstrated in this study.

#### Functional association of Oct4A with TUBB2A, PLEC and VIM in drug resistance models

**Figure 6:**
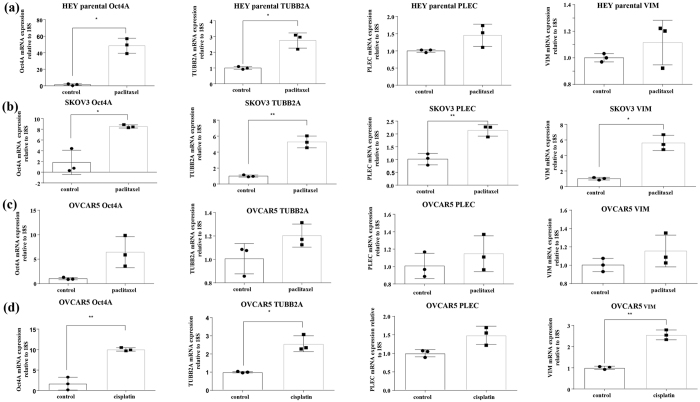
Expression of Oct4A, TUBB2A, PLEC and VIM in parental HEY, SKOV3 and OVCAR5 cell lines in response to paclitaxel or cisplatin treatment. (**a**) HEY, (**b**) SKOV3 and (**c,d**) OVCAR5 cell lines were treated with GI50 concentrations of paclitaxel or cisplatin for 72 hrs. RNA with and without paclitaxel or cisplatin treatments was extracted, cDNA was prepared and RT-PCR was performed as described in the Materials and Methods. The resultant mRNA levels were normalized to 18S mRNA. Results are representation of three independent experiments performed in triplicate. Significant variations between treated and untreated cells (control) was analysed by unpaired t-test with Welch’s correction using GraphPad Prism 7.02 and are indicated by *P < 0.05; **P < 0.01.

We have previously shown that the expression of Oct4/Oct4A is significantly enhanced in ovarian cancer cells in response to paclitaxel and cisplatin treatments^3^,^4^,^27^. In this study we demonstrate that the elevation of Oct4A at the mRNA level correlates with the enhancement in TUBB2A, PLEC and VIM expression in parental HEY, SKOV3 and OVCAR5 ovarian cancer cell lines in response to paclitaxel or cisplatin treatments (Fig. 6). This proof of concept observation consistently supports the functional association of Oct4A expression with key cytoskeletal/ECM associated proteins in ovarian cancer cell lines other than HEY cell line and suggests potential validity of this association in other tumor models.

## Discussion

We have previously demonstrated that suppression of Oct4A in the HEY cell line is sufficient to impact on OC tumorigenesis, metastasis and chemoresistance. Characteristics which were notably affected included cellular proliferation, adhesion, migration, invasion, increased sensitivity to chemotherapy treatment and overall decreased tumor initiating ability and metastasis in mouse models^27^,^34^. These diverse ranges of cellular traits affected by Oct4A knockdown strongly indicate direct or indirect regulation of Oct4A by signalling pathways and/or molecular mechanisms. In fact, clues as to how Oct4A influences these physiological changes within HEY cell line has come from previous observations that several markers associated with the CSC-like phenotype (Lin28, Sox2, CD44 and EpCAM), cellular adhesion and signalling integrins (α2, α5, α6, β1 and CD44), cellular invasion (Pro-MMP2), proliferation (Ki67), angiogenesis (CD31 and CD34) and survival (Bcl2 and GLUT1) were significantly impacted in HEY cells following knockdown of Oct4A expression^27^,^34^. However, these studies did not provide direct evidence of Oct4A specific proteome-mediated molecular mechanisms which influenced such an accumulative variety of biological processes. Therefore, we employed label-free MS-based proteomics to investigate the protein profiles between sample subsets (cell lysates and secretome and tumor xenografts) derived from HEY Oct4A KD and vector control cells to identify protein expression differences as a result of Oct4A regulation.

Overall, the data indicated Oct4A to be a key regulator of cytoskeleton/ECM remodelling besides its tumorigenic role that we have described previously^27^,^34^. Considering the extensive association of proteins identified, an effort was made to identify key proteins that regulate tumor-associated Oct4A traits in OC cells. Proteins which were consistently represented in the HEY Oct4A KD trait in each of the sample subsets were cytoskeleton proteins belonging to the tubulin, plakin and actin families. Of these TUBB2A, a member of the tubulin family and a major constituent of the cytoskeleton and critically involved in microtubule structure^36^ displayed concordant down regulation in the cellular (-42.9), xenograft (-78.1), and secretome (-8.0) subsets when Oct4A was down regulated. Besides TUBB2A, other members of tubulin family affected by Oct4A knockdown were TUBB6, TUBB2C, TUBB4A, TUBB4, TUBB5, which were prominently down regulated in Oct4A KD xenografts compared to xenografts derived from vector control cells.

More recently, TUBB2A has been described as having a role in regulating neuronal proliferation and migration^37^. Changes in β-tubulin isotype composition have been associated with tumor response to paclitaxel^38^,^39^ and increased tumor expression of β-tubulin II has been strongly associated with poor outcome in patients with head and neck carcinoma treated with docetaxel, a paclitaxel analogue^40^. Furthermore, increased TUBB2A expression has been correlated with decreased drug sensitivity in paclitaxel-resistant breast cancer cells^41^. This is consistent with our validation data which demonstrates a correlation of enhanced expression of TUBB2A with increased expression of Oct4A expression in paclitaxel or cisplatin treatment surviving resistant ovarian parental HEY, SKOV3 and OVCAR5 cell lines. Such evidence is also in agreement with our previous study, where we have demonstrated significant correlation between high Oct4A gene expression in recurrent OC patient’s ascites-derived tumor cells in response to treatment with a combination of cisplatin and paclitaxel compared to untreated (chemonaive) OC patient’s ascites derived tumor cells^27^.

Among other cytoskeleton proteins, PLEC a member of plakin family was significantly down regulated in Oct4A knockdown tumor xenograft and secretomes^42^. PLEC links the intermediate filament VIM with cytoplasmic organelles, and also provides stabilisation and links to nuclear envelope and centrosomes^43^. Increased PLEC and VIM expression, through PLEC-VIM complex has also been correlated to the migratory and invasive phenotypes in androgen-independent prostate and other cancers^44^,^45^,^46^. Loss of PLEC in HEY Oct4A KD cells coincides with the loss of VIM expression also seen in tumor xenografts. This is consistent with enhanced expression of PLEC and VIM in response to paclitaxel or cisplatin treatments in parental HEY, SKOV3 and OVCAR5 ovarian cell lines which coincided with elevated expression of Oct4A in these cells. This new finding adds novel clinical aspect of Oct4A regulation through key cytoskeletal and ECM proteins in the context of drug resistance, a major clinical hurdle for ovarian cancer patients.

The proteomic profiling of HEY Oct4A KD cells also demonstrated a loss of Mitogen activated protein kinase/Extracellular signal regulated kinase (MAPK/ERK) pathway in Oct4A knocked down tumor xenografts and secretomes. PLEC have been linked with MAPK/ERK pathway with respect to the migratory biology of keratinocytes and head and neck squamous cancer^46^,^47^. It was proposed that interaction of PLEC with MAPK/ERK pathway occurs due to interaction of PLEC with hemidesmosomal integrin α6β4, ligation of which by PLEC results in the activation of MAPK/ERK pathway^48^. Besides tubulin and plakins, actin-binding protein TAGLN2 involved with cancer cell motility and metastatic potential was also significantly decreased in HEY Oct4A KD samples^49^. These observations are consistent with decreased motility and metastatic potential of Oct4A KD cells that we have previously demonstrated^27^. On the other hand, actin-binding protein PLS1 and TWF1 involved with cell motility and mitotic division were significantly increased in HEY Oct4A KD samples. This increase in actin-binding PLS1 and TWF1^50^,^51^ may occur to compensate the decrease in cytoskeleton tubulins, plakins and TAGLN. However, this compensatory increase may not provide requisite level of signals to initiate the migratory and metastatic potentials in the absence of Oct4A signals.

We have recently shown that suppression of Oct4A in HEY cell line resulted in the loss of αv and α2 family of integrins^34^. This observation is consistent with the proteomics data in the current study which showed a loss of αv and α2 family of integrins and integrin-linked kinase (ILK) in the secretomes and tumor xenografts derived from HEY Oct4A KD cells compared to vector control cells derived secretomes and xenografts. ILK has previously been shown to regulate mitotic cytoskeleton dynamics in retinoblastomas^52^. Consistent with that we see loss of Fibronectin (FN) and Laminin (LM) in the secretomes, and only loss of FN in the tumor xenografts derived from Oct4A KD cells compared to that derived from vector control cells. As FN and LM form important constituents of ovarian ECM and has significant roles in ovarian tumorigenesis^53^,^54^, it is likely that the loss of FN and LM in combination with cytoskeletal PLEC, VIM and integrins contributes to the loss of tumorigenic and invasive potential of Oct4A KD cells in mouse models as reported in our previous studies^27^.

Protein secretion by ovarian tumor cells has been shown to result in autocrine and paracrine signalling that defines cell growth, migration and the makeup of extracellular environment^55^. Secretion of PLEC in extracellular cyst fluid has been identified as a biomarker for the detection of early intra-ductal papillary mucinous neoplasms, a group of lesions with varying metastatic potential often detectable by CT scan^56^. PLEC expression increases during the development of pancreatic intraepithelial neoplasia, pre-cursor lesions of invasive and metastatic pancreatic ductal adenocarcinoma (PDAC)^57^. In addition, secretory form of PLEC makes an important component of exosomes of pancreatic cancer cells where it couples with α6β4 integrin^58^. In contrast, pericellular FN has also been shown to promote the metastasis of lung cancer cells by adhering to the cell surface receptor dipeptidyl peptidase IV (DPP IV)^59^. Hence, loss of secretory FN and PLEC in response to Oct4A KD may contribute to loss of migration, *in vivo* invasion and tumor development that we have reported previously^34^.

Proteins found to be up regulated in HEY Oct4A KD were primarily associated with cellular survival. These categories included cellular proliferation, lipid metabolism, cellular metabolism, cellular growth and oxidative stress. This included cytoskeleton (e.g. TWF1), cellular growth (EIF42A), cellular metabolism (IDH2) and oxidative stress (HBA1). Interestingly, despite the up regulation of these proteins, HEY Oct4A KD cells derived tumors displayed an overall reduced growth and tumorigenic potential in mouse models^27^,^34^. This may simply be due to the fact that up regulated proteins were not as strongly represented in HEY Oct4A KD samples compared to down regulated proteins. Logically, down regulated proteins may have a stronger influence on the phenotype of HEY Oct4A KD cells. However, it may also be that proteins which were up regulated are done as a compensatory mechanism for the stressful changes occurring in the overall phenotype of the HEY Oct4A KD cells. For instance, increased oxidative stress is a phenomenon shown to occur as a result to increased cellular metabolism^60^. Hence, cellular metabolism and oxidative stress proteins are strongly represented as up regulated in HEY Oct4A KD proteins. This may suggest oxidative stress response proteins are being produced to compensate the elevated cellular metabolism occurring in Oct4A KD cells to sustain their survival. An altered cellular metabolism has been shown to occur in CSCs^61^,^62^. However, it remains unclear why HEY Oct4A KD cells undergo increased cellular metabolism and requires further analysis. However, proteins which appear to be involved in cellular apoptosis (e.g. POTEF) and tumor suppression (APOA1) were also noted to be up regulated following Oct4A suppression. This indicates that there may be some strong influence of pro-survival mechanisms in HEY cells following suppression of Oct4A expression. However, the up regulated proteins involved with cellular apoptosis and tumor suppression may have positive implications in reducing tumor growth and survival of HEY cells and may support the reduced tumor growth observed previously in HEY Oct4A KD cells both *in vitro* and *in vivo*^27^,^34^.

## Conclusion

This study has for the first time identified the global proteome profile associated with Oct4A in ovarian cancer, targeting cellular, secretome and tumor xenograft subsets. These findings along with the validation of key cytoskeletal and ECM proteins (TUBB2A, PLEC, VIM) support the diminutive changes of proteins associated with cytoskeleton and ECM as major targets of Oct4A knockdown. In addition, gene/protein data using ICGC (International Cancer Genome Consortium, http://icgc.org), EBI Expression Atlas (http://www.ebi.ac.uk) and The Cancer Genome Atlas (https://gdc-portal.nci.nih.gov) which focused on ovarian tumors/tissues, suggest over expression of Oct4 in ovarian tumors. Furthermore, Oncomine (A Cancer Microarray Database and Integrated Data, https://www.oncomine.com/) indicates increased Oct4 gene expression (fold change 6.313) in ovarian tumors in comparison to normal ovarian tissues. Even though VIM/PLEC/TUBB2A demonstrated high expression in ovarian tumors (top 1%), no comparison with normal ovarian tissue exists in these databases so no enrichment/fold change can be indicated in tumors. These data support an association of Oct4A with cytoskeletal-ECM network, the key findings of this large-scale proteomics study. The attenuation of tumorigenic phenotype of HEY cells resulting from the knock down of Oct4A shown in our previous study further support these findings^27^,^34^.

**Figure 7:**
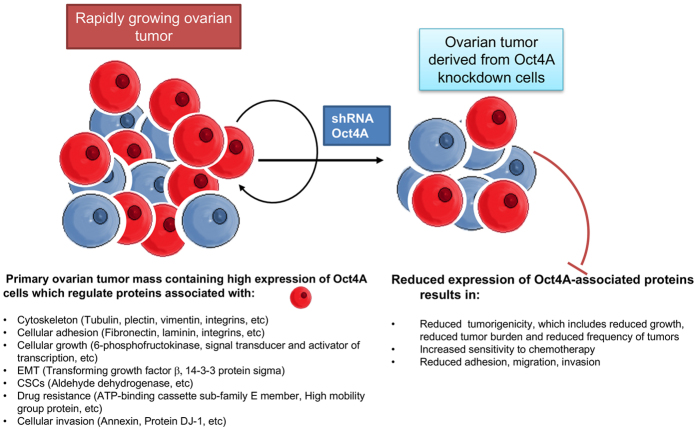
Proposed model of Oct4A regulation in ovarian carcinomas. Ovarian tumors contain high expression of Oct4A which promotes ovarian tumorigenesis^27^. Knockdown of Oct4A in ovarian cancer cells results in the down regulation of major regulatory network of proteins including those involved with cytoskeleton-ECM remodelling, proliferation and cellular growth, EMT, CSCs, cellular adhesion, drug resistance and cellular invasion. This promotes the diminution of tumorigenic phenotypes which we have shown in our previous studies^13^,^27^,^34^.

These observations are sustained in drug resistant models where up regulation of Oct4A in paclitaxel or cisplatin treatment surviving residual parental HEY, SKOV3 and OVCAR5 cell lines correlated with the up regulation of TUBB2A, PLEC and VIM expression. This is consistent with the network pathway analyses which identified cytoskeleton and ECM proteins to be significantly diminished in the target subsets. However, network pathway analyses also identified clusters of proteins regulating glycolysis(Glyceraldehyde-3-phosphate dehydrogenase (GAPDH), Lactate dehydrogenase live type A (LDHA), Phospho fructo-kinase (PFK), Pyruvate dehydrogenase (PDH)) and (fatty acid synthesisFatty acid synthase (FASN)) to diminish in response to Oct4A knockdown. Overall, the protein changes observed are highly complex but the networks and results of this current proteomic analysis support the findings of our previous studies performed *in vitro* and *in vivo*^13^,^27^,^34^. The identified proteins described in this study have a strong implication in understanding Oct4A related functions in ovarian tumors in general and may apply in understanding Oct4A related functions in tumor models of other cancers. Based on this proteomic analysis a model of Oct4A regulation in ovarian carcinomas has been proposed in Fig. 7.

## Declarations

### Ethics Approval

#### Animal ethics statement

This study was carried out in strict accordance with the recommendations in the Guide for the Care and Use of the Laboratory Animals of the National Health and Medical Research Council of Australia. The experimental protocol was approved by the Ludwig Institute/Department of Surgery, Royal Melbourne Hospital and University of Melbourne’s Animal Ethics Committee (Project-006/11), and was endorsed by the Research and Ethics Committee of Royal Women’s Hospital Melbourne, Australia.

## Additional Information

**How to cite this article**: Samardzija, C. *et al*. Knockdown of stem cell regulator Oct4A in ovarian cancer reveals cellular reprogramming associated with key regulators of cytoskeleton-extracellular matrix remodelling. *Sci. Rep.* **7**, 46312; doi: 10.1038/srep46312 (2017).

**Publisher's note:** Springer Nature remains neutral with regard to jurisdictional claims in published maps and institutional affiliations.

## Supplementary Material

Supplementary Dataset

Supplementary References in Tables

## Figures and Tables

**Table 1:** Human oligonucleotide primer sequences for quantitative real-time PCR.

Gene Symbol	Accession no.	Primer sequences from 5′-3′	Size (bp)
*Rn18S*	NR_003286.1	Forward GTAACCCGTTGAACCCCATTReverse CCATCCAATCGGTAGTAGCG	153
*Oct4A*	NM_002701.4	Forward CTCCTGGAGGGCCAGGAATCReverse CCACATCGGCCTGTGTATAT	381
*VIM*	NM_003380	Forward CCTACAGGAAGCTGCTGGAAReverse GGTCATCGTGATGCTGAGAA	198
*PLEC*	NM_201384	Forward TACTACCGCGAGAGTGCAGAReverse TCCTTGATGGCGTTGATGTA	212
*TUBB2A*	NM_001069.2	Forward CTTCGGCCAGATCTTCAGACReverse GAGAGTGGGTCAGCTGGAAG	176

**Table 2:** Down regulated cellular proteins in HEY Oct4A KD cells according to cellular function.

Category	Gene Name	Protein Description	Rsc	*Reference
Cytoskeletal	TUBB2A	Tubulin beta-2A chain	-42.9	(McCarroll and Kavallaris, 2012)
	ACTC1	Alpha-cardiac actin	-1.7	(Tondeleir *et al*., 2011)
	ACTB	Beta-actin	-1.5	(Guo *et al*., 2013)
	PLEC	Plectin	-1.2	(Katada, K., *et al*., 2012)
Epithelial-Mesenchymal Transition	YWHAE	14-3-3ε	-10.0	(Liu *et al*., 2013a)
	PPP2CA	Serine/threonine-protein phosphatase 2A catlytic subunit alpha isolform	-3.3	(Bhardwaj *et al*., 2014)
	VIM	Vimentin	-1.2	(Mendez *et al*., 2010)
Cellular metabolism	ECHS1	Enoyl-CoA Hydratase 1	-3.7	(Carracedo *et al*., 2013)
	SLC25A3	Solute carrier damily 25 member 3	-2.4	(Palmieri, 2013)
	CS	Citrate synthase	-2.4	(Gaude and Frezza, 2014)
Cellular migration/motility	EFHD2	Swiprosin-1	-4.1	(Huh *et al*., 2015)
	PFN1	Profilin-1	-1.8	(Ding *et al*., 2012)
Drug resistance	GLO1	Glyoxalase 1	-3.1	(Sakamoto *et al*., 2000)
	TAGLN2	Transgelin-2	-3.0	(Chen *et al*., 2014)
Protein transport	SEC63	Translocation protein SEC63 homolog	-3.4	(Zimmermann *et al*., 2006)
Cell adhesion	RSU1	Ras suppressor protein 1	-2.8	(Kim *et al*., 2015b)
Immune response	WARS	Tryptophan-tRNA ligase,	-2.8	(Mellor and Munn, 1999)
Prognosticator of survival	ALB	Serum albumin	-1.8	(Gupta and Lis, 2010)
Post-translational modifications	HIST1H4B	Histone H4	-1.6	(van der Meijden *et al*., 1998)

Rsc: Protein abundance ratio (HEY Oct4A KD/HEY vector control).

*Supplementary File 1.

**Table 3:** Up regulated cellular proteins in HEY Oct4A KD cells.

Category	Gene Name	Protein Description	Rsc	*Reference
Cytoskeletal	PLS1	Plastin-1	7.1	(Delanote *et al*., 2005)
	TWF1	Twinfilin-1	5.1	(Moseley *et al*., 2006)
	EPB41L2	Erythrocyte membrane protein band 4.1-like 2	4.5	(Lu *et al*., 2004)
Tumour suppression	APOA1	Apolipoprotein A-1	5.1	(Zamanian-Daryoush *et al*., 2013)
	ETF1	Eukaryotic release factor 1	2.3	(Dubourg *et al*., 2002)
	AHNAK	Desmoyokin	1.8	(Lee *et al*., 2014)
Cellular growth/survival	TFRC	Transferrin receptor protein 1 (CD71)	2.9	(Habashy *et al*., 2010)
	DDX3X	ATP-dependant RNA helicase DDX3X	2.6	(Lai *et al*., 2010)
	EIF4A2	Eukaryotic initiation factor 4A2	1.8	(Modelska *et al*., 2015)
Drug resistance	TYMS	Thymidylate synthase (EC 2.1.1.45)	5.1	(Wang *et al*., 2007)
	ATP1A1	Sodium pump subunit alpha-1	1.8	(Stordal *et al*., 2012)
Lipid metabolism	NCEH1	Neutral cholestrol ester hydrolase 1	3.8	(Chiang *et al*., 2006)
	PRIC295	Peroxisome proliferator activated receptor interacting complex protein	2.6	(Pyper *et al*., 2010)
Extracellular matrix	PLOD2	Procollagen-lysine, 2-oxoglutarate 5-dioxygenase 2	2.1	(Gilkes *et al*., 2013)
Drug sensitivity	PEBP1 (RKIP)	Phosphatidylethanolamine-binding protein 1	1.9	(Li *et al*., 2014a)
Immune response	FLNC	Filamin-C	1.4	(Marti *et al*., 1997)

Rsc: Protein abundance ratio (HEY Oct4A KD2/HEY vector control).

*Supplementary File 1.

**Table 4:** Down regulated secreted proteins in HEY Oct4A KD cells.

Category	Gene Name	Protein Description	Rsc	*Reference
Cytoskeletal	TUBB2A	Tubulin beta-2A chain (Tubulin beta class IIa)	-8.0	(McCarroll and Kavallaris, 2012)
	APLP2	Amyloid-like protein 2	-5.8	(Pandey *et al*., 2015)
	RDX	Radixin	-5.6	(Hoeflich and Ikura, 2004)
	FLNB	Filamin-B	-5.0	(Popowicz *et al*., 2006)
	FLNA	Filamin-A	-4.5	(Popowicz *et al*., 2006)
	ACTN2	Alpha-actinin-2	-4.5	(Djinovic-Carugo *et al*., 2002)
Cellular growth/survival	UBA1	Ubiquitin-like modifier-activating enzyme 1	-5.6	(Moudry *et al*., 2012)
	EIF4A2	Eukaryotic initiation factor 4A-II	-4.7	(Modelska *et al*., 2015)
	XPO1	Exportin-1	-3.6	(van der Watt *et al*., 2009)
	CTSD	Cathepsin D (EC 3.4.23.5)	-3.4	(Langhoff *et al*.)
	CLTC	Clathrin heavy chain 1	-2.2	(McMahon and Boucrot, 2011)
Extracellular matrix	FN1	FN1 protein (Fibronectin 1)	-15.3	(Singh *et al*., 2010)
	LAMC2	Laminin subunit gamma-2	-10.2	(Garg *et al*., 2014)
	LAMB3	Laminin subunit beta-3	-7.8	(Aumailley, 2013)
	APP A4	Amyloid beta A4 protein	-3.2	(Klier *et al*., 1990)
Drug resistance	FASN	Fatty acid synthase (EC 2.3.1.85)	-5.3	(Wu *et al*., 2014)
	TAGLN2	Transgelin-2	-4.7	(Chen *et al*., 2014)
	PSAT	Phosphoserine aminotransferase (EC 2.6.1.52)	-3.4	(Vie *et al*., 2008)
Cellular adhesion	PPIB	Peptidyl-prolyl cis-trans isomerase B (cyclophilin B)	-4.5	(Melchior *et al*., 2008)
	FN1 FN	Fibronectin (Cleaved into: Anastellin)	-2.9	(Mercurius and Morla, 2001)
	VCL	Vinculin (Metavinculin)	-2.8	(Demali, 2004)
Cancer stem cells	CD109	CD109 antigen	-5.0	(Emori *et al*., 2013)
	ANPEP	Aminopeptidase N	-2.8	(Kim *et al*., 2012a)
Immune response	LTA4H	Leukotriene A-4 hydrolase	-4.7	(Chen *et al*., 2004)
	AEBP1	Adipocyte enhancer-binding protein 1	-3.2	(Holloway *et al*., 2012)
Cellular invasion	PLEC	Plectin	-6.7	(Katada *et al*., 2012)
Angiogenesis	TIE1	Tyrosine-protein kinase receptor Tie-1 (EC 2.7.10.1)	-3.4	(Jones *et al*., 2001)
Epithelial-Mesenchymal Transition	DRIP4	Dopamine receptor interacting protein 4	-2.8	(Ji *et al*., 2015)

Rsc: Protein abundance ratio (HEY Oct4A KD2/HEY vector control).

*Supplementary File 1.

**Table 5:** Up regulated secreted proteins in HEY Oct4A KD cells.

Category	Gene Name	Protein Description	Rsc	*Reference
Cytoskeletal	TUBB2C	Tubulin beta-2C	2.3	(McCarroll and Kavallaris, 2012)
	TUBB4A	Tubulin beta-4 chain	2.5	(McCarroll and Kavallaris, 2012)
	TUBB5	Tubulin beta-5 chain	1.7	(McCarroll and Kavallaris, 2012)
	TUBB6	Tubulin beta-6 chain	2.1	(McCarroll and Kavallaris, 2012)
	ACTC1	Actin, alpha cardiac muscle 1	2.3	(Tondeleir *et al*., 2011)
	ACTB	Beta Actin	2.3	(Guo *et al*., 2013)
Tumour suppression	HBB	Mutant beta-globin	5.0	(Onda *et al*., 2005)
	APOA1	Apolipoprotein A1	4.3	(Zamanian-Daryoush *et al*., 2013)
	ITIH3	Inter-alpha-trypsin inhibitor heavy chain H3	3.3	(Paris *et al*., 2002)
	ITIH2	Inter-alpha (Globulin) inhibitor H2	3.1	(Hamm *et al*., 2008)
	A2M	Alpha-2-macroglobulin	3.3	(Lindner *et al*., 2010)
Cellular growth/survival	HSP90AA1	Heat shock protein HSP 90-alpha	2.0	(Chu *et al*., 2013)
	SERPINE1	Plasminogen activator inhibitor	1.8	(Gomes-Giacoia *et al*., 2013)
	HSPA8	Heat shock cognate 71 kDa protein	1.6	(Rohde *et al*., 2005)
	HSP90AB1	Heat shock protein HSP 90-beta	1.5	(Haase and Fitze, 2015)
Oxidative stress response	GSTP1	Glutathione S-transferase P	3.3	(Kanwal *et al*., 2014)
	HPX	Hemopexin	2.9	(Tolosano and Altruda, 2002)
	HBA1	Hemoglobin subunit alpha	2.7	(Li *et al*., 2013c)
Prognosticator of survival	ALB	Serum albumin	4.3	(Gupta and Lis, 2010)
	AFP	Alpha-fetoprotein	3.1	(Li *et al*., 2013a)*
Immune response	PTX3	Pentraxin-related protein PTX3	4.1	(Bonavita *et al*., 2015)
	C4A	Complement C4-A	2.7	(Pio *et al*., 2013)
Cellular invasion	ANXA1	Annexin A1	4.7	(Cheng *et al*., 2012)
	ANXA2	Annexin A2	3.3	(Lokman *et al*., 2013)
Estrogen-induced malignancy	SERPINA7	Thyroxine-binding globulin	4.7	(Doe *et al*., 1967)
Angiogenesis	APOB	Apolipoprotein B-100	3.9	(Avraham-Davidi *et al*., 2012)*
Apoptosis	POTEF	POTE ankyrin domain family member F	2.6	(Liu *et al*., 2009)
Iron transport	TF	Transferrin	2.5	(Kovac *et al*., 2011)

Rsc: Protein abundance ratio (HEY Oct4A KD2/HEY vector control).

*Supplementary File 1.

**Table 6:** Down regulated proteins in HEY Oct4A KD tumor xenografts.

Category	Gene Name	Protein Description	Rsc	*Reference
Cellular growth	BCAT1	Branched chain amino acid aminotransferase	-10.1	(Wang *et al*., 2015b)
	PPP1CB	Serine/threonine-protein phosphatase PP1-beta subunit	-7.4	(Velusamy *et al*., 2013)
	MTHFD1L	Monofunctional C1-tetrahydrofolate synthase	-5.6	(Tedeschi *et al*., 2013)
	RPL13	60S ribosomal protein L13	-4.7	(Kobayashi *et al*., 2006)
	CAP1 hCG_2033246	Adenylyl cyclase-associated protein	-4.6	(Hua *et al*., 2015)
	TMPO (LAP2)	Lamina-associated polypeptide 2, isoform alpha Thymopoietin isoform alpha	-3.2	(Brachner and Foisner, 2014)
	CLTC	Calathrin heavy chain 2	-2.7	(McMahon and Boucrot, 2011)
	HSPA1A	Heat shock 70 kDa protein 1A/1B	-1.3	(Wu *et al*., 2012)
	HSP90AB1	Heat shock protein HSP 90-beta	-1.3	(Haase and Fitze, 2015)
	PABPC4	Poly(A) binding protein 4	-1.3	(Katzenellenbogen *et al*., 2010)
	HIST1H2BD	Histone H2B type 1-D	-1.3	(Maruyama *et al*., 2014)
Cellular invasion	PDLIM1	LIM domain protein 1	-10.1	(Liu *et al*., 2015c)
	FAM49B	Protein FAM49B	-7.6	(Sung *et al*., 2014)
	DPYSL3	Dihydropyrimidinase-related protein 3	-6.5	(Hiroshima *et al*., 2013)
	GNAI3	Guanine nucleotide-binding protein G subunit alpha 3	-4.7	(Zhang *et al*., 2015)*
	PLEC	Plectin	-3.2	(Katada *et al*., 2012)
	ANXA6	Annexin A6	-2.9	(Sakwe *et al*., 2011)*
	CA3	Carbonic anhydrase 3 (EC 4.2.1.1) (Carbonic anhydrase III) (CA-III)	-1.7	(Dai *et al*., 2008a)
	ANXA2	Annexin A2	-1.6	(Lokman *et al*., 2013)
	ALDOA	Fructose-bisphosphate aldotase A	-1.4	(Sun *et al*., 2014)
	ANXA1	Annexin A1	-1.4	(Cheng *et al*., 2012)
	FKBP1A	Peptidyl-prolyl cis-trans isomerase	-1.1	(Fong *et al*., 2003)
Drug resistance	CAPN2	Calpain-2	-9.1	(Storr *et al*., 2012)
	DYNC1H1	Cytoplasmic dynein 1 heavy chain 1	-8.5	(Huang *et al*., 2014)
	PSMD1	26S proteasome regulatory subunit RPN2	-7.4	(Honma *et al*., 2008)
	PRKDC	DNA-dependent protein kinase catalytic subunit	-3.9	(Helleday *et al*., 2008)
	TGM2	Protein -glutamine gamma-glutamyltransferase 2	-2.7	(Cao *et al*., 2008)
	HMGB1	High mobility group protein B1	-1.7	(Huang *et al*., 2012a)
	PEBP1(RKIP)	Phosphatidylethanolamine-binding protein 1	-1.7	(Liu *et al*., 2015a)
	MDH2	Malate dehydrogenase	-1.5	(Lo *et al*., 2015)
	HSP90AA1	Heat shock protein HSP 90-alpha	-1.3	(Chu *et al*., 2013)
Apoptosis	RLI	RNase L inhibitor	-8.1	(Li *et al*., 2014b)
	SLC25A6	ADP/ATP translocase 3 (ANT3)	-7.4	(Yang *et al*., 2007)
	LRPPRC	Leucine-rich PPR motig containing protein	-5.1	(Zhou *et al*., 2014)*
	SMC3	Structural maintenance of chromosome 3	-4.7	(Ghiselli, 2006)*
	COPA	Coatomer subunit alpha	-4.6	(Sudo *et al*., 2010)*
	MAGED2	Melanoma-associated antigen D2	-3.8	(Tseng *et al*., 2012)*
	NT5E (CD73)	5′-nucleotidase	-1.3	(Zhi *et al*., 2010)*
Cancer stem cells	CD109	Cluster of differentiation 109	-12.8	(Emori *et al*., 2013)
	PDHB	Pyruvate dehydrogenase E1 component subunit beta	-11.9	(Anderson *et al*., 2014)
	ALDH18A1	Aldehyde dehydrogenase family 18 member A1	-3.8	(Buijs *et al*., 2012)
	NES hCG_1999207	Nestin isoform CRA	-1.1	(Neradil and Veselska, 2015)
Cytoskeletal	TUBB2A	Tubulin beta-2A chain	-78.1	(McCarroll and Kavallaris, 2012)
	CD2AP	Adaptor protein CMS	-6.5	(Lynch *et al*., 2003)
	TUBB4A	Tubulin beta 4 A chain	-1.9	(McCarroll and Kavallaris, 2012)
	TUBB5	Tubulin beta 5 chain	-1.7	(McCarroll and Kavallaris, 2012)
Cellular adhesion	FN1 FN	Fibronectin (Cleaved into: Anastellin)	-55.4	(Mercurius and Morla, 2001)
	ILK	Integrin-linked protein kinase	-5.6	(Wang and Basson, 2009)
	ITGA2	Integrin alpha-2	-2.9	(Van Slambrouck *et al*., 2009)
Cellular metabolism	HIST2H2BE	Histone H2B type 2-E	-46.3	(Dai *et al*., 2008b)
	PFKP	Phosphofructo-1-kinase isozyme C	-10.1	(Moon *et al*., 2011)
	YARS2	Tyrosyl tRNA synthetase	-3.8	(Riley *et al*., 2010)
Epithelial-Mesenchymal Transition	TGFB1	Transforming growth factor-beta-induced protein	-12.8	(Xu *et al*., 2009a)
	HNRNPM	Heterogeneous ribonucleoprotein M	-5.8	(Xu *et al*., 2014)
	PLS3	Plastin-3	-4.7	(Sugimachi *et al*., 2014)
Transcriptional regulation	RUVBL2	RuvB-like 2 (EC 3.6.4.12) (48 kDa TATA box-binding protein-interacting protein)	-7.4	(Flavin *et al*., 2011)
	COPS7A	Signalosome subunit 7a	-5.6	(Singer *et al*., 2014)
	HNRNPD	Heterogeneous nuclear ribonucleoprotein D0 (hnRNP D0) (AU-rich element RNA-binding protein 1)	-1.5	(Moore *et al*., 2014)
DNA repair	RECQL	ATP-dependant DNA helicase Q1	-11.0	(Kawabe *et al*., 2000)
	UBA1	Ubiquitin-activating enzyme E1	-1.1	(Moudry *et al*., 2012)
Tumour suppression	MAP1B	Microtubule-associated protein 1B	-5.6	(Lee *et al*., 2008)*
	AHNAK	Desamoyokin	-3.5	(Lee *et al*., 2014)*
	OGDH	2-oxoglutarate dehydrogenase	-1.1	(Tennant and Gottlieb, 2010)
Angiogenesis	TXNRD1	Thioredoxin reductase 1	-4.7	(Welsh *et al*., 2002)
	AARS	Alanine tRNA ligase	-4.7	(Mirando *et al*., 2015)
Protein transport	VPS35	Vacuolar protein sorting-associated protein 35	-3.0	(Seaman *et al*., 1997)
	EEA1	Early endosome antigen 1	-2.2	(Christoforidis *et al*., 1999)
Extracellular matrix	FN1	Fibronectin 1 Protein	-57.2	(Singh *et al*., 2010)
Carcinogen detoxification	CYB5R3	NADH-cytochrome b5 reductase 3 (Diaphorase-1)	-14.6	(Kurian *et al*., 2006)
Immune Response	LTA4H	Leukotriene A-4 hydrolase	-10.6	(Chen *et al*., 2004)
Cellular migration/motility	KTN1	KTN1 protein (highly similar to Kinectin)	-5.6	(Zhang *et al*., 2010b)
Unknown function	TMPO hCG_2015322	Thymopentin isoform CRA_d	-2.7	Peer reviewed information could not be found

Rsc: Protein abundance ratio (HEY Oct4A KD2/HEY vector control).

*Supplementary File 1.

**Table 7:** Up regulated proteins in HEY Oct4A KD tumor xenografts.

Category	Gene Name	Protein Description	Rsc	*Reference
Cellular metabolism	GOT1	Aspartate aminotransferase	2.0	(Lyssiotis *et al*., 2013)
	PYGB	Phosphorylase (EC 2.4.1.1)	1.5	(Willmann *et al*., 2015)
	GOT2	Glutamic-oxaloacetic transaminase 2	1.1	(Lyssiotis *et al*., 2014)
	IDH2	Isocitrate dehydrogenase	2.5	(Borodovsky *et al*., 2012)
	ATP5O	ATP synthase subunit O	1.3	(Antoniel *et al*., 2014)
Cellular growth	EIF42A	Eukaryotic translation initiation factor 4A2	5.9	(Modelska *et al*., 2015)
	CKB	Creatine kinase B-type	3.7	(Li *et al*., 2013b)
	PCBP2 hCG_2017557	Poly(RC) binding protein 2	1.3	(Hu *et al*., 2014a)
	TOMM34	Mitochondrial import receptor subunit TOM34	1.3	(Shimokawa *et al*., 2006)
Apoptosis	POTEF	POTE ankyrin domain family member F	23.0	(Liu *et al*., 2009
	HSPH1	Heat shock protein 105 kDa	1.5	(Kennedy *et al*., 2014)
	HNRNPH3	Heterogeneous nuclear ribonucleoprotein H3	1.4	(Garneau *et al*., 2005)
	COMT	Catechol-*O*-methyltransferase	1.1	(Wu *et al*., 2015b)
Tumour suppression	AMY2A	Alpha amylase	7.3	(Kang *et al*., 2010)
	HBB	Mutant beta-globin	1.9	(Onda *et al*., 2005)
	KRBA2	KRAB-A domain-containing protein	1.7	(Li *et al*., 2003)
	TPM1	Tropomyosin-1	1.2	(Bharadwaj and Prasad, 2002)
Oxidative stress response	NUDT5	ADP-sugar pyrophosphatase	3.1	(McLennan, 2006)
	DNAJB11	DnaJ homolog subfamily B member 11	1.3	(Nakanishi *et al*., 2004)
	HBA1	Alpha-globin	1.2	(Li *et al*., 2013c)
	KPNA3	Karyopherin subunit alpha-3	1.0	(Young *et al*., 2013)
Cytoskeletal	NEB	Nebulin	1.9	(Pappas *et al*., 2011)
	TPM2	Tropomyosin-2	1.1	(Assinder *et al*., 2010)
Calcium homeostasis	ATP2A1	Sarcoplasmic/endoplasmic reticulum calcium ATPase 1	5.6	(Arbabian *et al*., 2011)
	ATP2A2	Sarcoplasmic/endoplasmic reticulum calcium ATPase 2	4.1	(Arbabian *et al*., 2011)
Prognosticator of survival	ALB	Serum albumin	3.7	(Gupta and Lis, 2010)
	HP	Haptoglobin (Zonulin)	3.0	(Zhao *et al*., 2007)*
Drug resistance	COL6A3	Collagen alpha-3(VI) chain	3.7	(Chen *et al*., 2013b)
	TPI1	Triosephosphate isomerase 1	1.0	(Wang *et al*., 2008)*
Iron transport	TF	Transferrin	3.7	(Kovac *et al*., 2011)
Angiogenesis	FMOD	Fibromodulin	3.7	(Jian *et al*., 2013)
Cancer stem cells	ANPEP	Aminopeptidase N	1.1	(Kim *et al*., 2012a)
mRNA regulation	NHP2L1	NHP2-like protein 1	2.1	(Esteller, 2011)
Cellular adhesion	PRELP	Prolargin	2.0	(Bengtsson *et al*., 2002)
Protein synthesis	RPS17L	40S ribosomal protein S17	1.7	(Chen and Roufa, 1988)
Immune response	FLNC	Filamin-C	1.3	(Marti *et al*., 1997)
Cellular migration/motility	BGN	Biglycan	1.2	(Hu *et al*., 2014b)

Rsc: Protein abundance ratio (HEY Oct4A KD2/HEY vector control).

*Supplementary File 1.
