# Supplementary material for: Knockdown of stem cell regulator Oct4A in ovarian cancer reveals cellular reprogramming associated with key regulators of cytoskeleton-extracellular matrix remodelling
Source: Sci Rep. 2017 Apr 13;7:46312. doi: 10.1038/srep46312 (PMC5390261; doi:10.1038/srep46312)
Supplement: Supplementary References in Tables [file srep46312-s2.pdf]

References in Tables (1-7):

Manuscript title: Knockdown of stem cell regulator Oct4A in ovarian cancer reveals cellular reprogramming associated with key regulators of cytoskeleton-extracellular matrix remodelling

Authors: Chantel Samardzija, David W Greening[2], Ruth Escalona, Maoshan Chen, Maree Bilandzic, Rodney Luwor, George Kannourakis, Jock K Findlay and Nuzhat Ahmed

1. Anderson, A.S., et al., Ovarian tumor-initiating cells display a flexible metabolism. Experimental Cell Research, 2014. 328(1): p. 44-57.
2. Antoniel, M., et al., The Oligomycin-Sensitivity Conferring Protein of Mitochondrial ATP Synthase: Emerging New Roles in Mitochondrial Pathophysiology. International Journal of Molecular Sciences, 2014. 15(5): p. 7513-7536.
3. Arbabian, A., et al., Endoplasmic reticulum calcium pumps and cancer. Biofactors, 2011. 37(3): p. 139-49.
4. Assinder, S.J., et al., A novel splice variant of the beta-tropomyosin (TPM2) gene in prostate cancer. Mol Carcinog, 2010. 49(6): p. 525-31.
5. Aumailley, M., The laminin family. Cell Adh Migr, 2013. 7(1): p. 48-55.
6. Avraham-Davidi, I., et al., ApoB-containing lipoproteins regulate angiogenesis by modulating expression of VEGF receptor 1. Nature Medicine, 2012. 18(6): p. 967-973.
7. Bengtsson, E., et al., The leucine-rich repeat protein PRELP binds perlecan and collagens and may function as a basement membrane anchor. J Biol Chem, 2002. 277(17): p. 15061-8.
8. Bharadwaj, S. and G.L. Prasad, Tropomyosin-1, a novel suppressor of cellular transformation is downregulated by promoter methylation in cancer cells. Cancer Lett, 2002. 183(2): p. 205-13.
9. Bhardwaj, A., et al., Restoration of PPP2CA expression reverses epithelial-to-mesenchymal transition and suppresses prostate tumour growth and metastasis in an orthotopic mouse model. Br J Cancer, 2014. 110(8): p. 2000-10.
10. Bonavita, E., et al., PTX3 Is an Extrinsic Oncosuppressor Regulating Complement-Dependent Inflammation in Cancer. Cell, 2015. 160(4): p. 700-714.
11. Borodovsky, A., M.J. Seltzer, and G.J. Riggins, Altered cancer cell metabolism in gliomas with mutant IDH1 or IDH2. Current Opinion in Oncology, 2012. 24(1): p. 83-89.
12. Brachner, A. and R. Foisner, Lamina-associated polypeptide (LAP)2? and other LEM proteins in cancer biology. Advances In Experimental Medicine And Biology, 2014. 773: p. 143-163.
13. Buijs, J.T., et al., The BMP2/7 heterodimer inhibits the human breast cancer stem cell subpopulation and bone metastases formation. Oncogene, 2012. 31(17): p. 2164-74.

14. Cao, L., et al., Tissue transglutaminase protects epithelial ovarian cancer cells from cisplatin-induced apoptosis by promoting cell survival signaling. Carcinogenesis, 2008. 29(10): p. 1893-900.

15. Carracedo, A., L.C. Cantley, and P.P. Pandolfi, Cancer metabolism: fatty acid oxidation in the limelight. Nat Rev Cancer, 2013. 13(4): p. 227-232.

16. Chen, I. and D.J. Roufa, The transcriptionally active human ribosomal protein S17 gene. Gene, 1988. 70(1): p. 107-116.

17. Chen, P., M. Cescon, and P. Bonaldo, Collagen VI in cancer and its biological mechanisms. Trends in Molecular Medicine, 2013. 19(7): p. 410-417.

18. Chen, S., et al., Proteomic analysis of the proteins that are associated with the resistance to paclitaxel in human breast cancer cells. Mol Biosyst, 2014. 10(2): p. 294-303.

19. Chen, X., et al., Leukotriene A4 hydrolase as a target for cancer prevention and therapy. Curr Cancer Drug Targets, 2004. 4(3): p. 267-83.

20. Cheng, T.Y., et al., Annexin A1 is associated with gastric cancer survival and promotes gastric cancer cell invasiveness through the formyl peptide receptor/extracellular signal-regulated kinase/integrin beta-1-binding protein 1 pathway. Cancer, 2012. 118(23): p. 5757-67.

21. Chiang, K.P., et al., An enzyme that regulates ether lipid signaling pathways in cancer annotated by multidimensional profiling. Chem Biol, 2006. 13(10): p. 1041-50.

22. Christoforidis, S., et al., The Rab5 effector EEA1 is a core component of endosome docking. Nature, 1999. 397(6720): p. 621-5.

23. Chu, S.-H., et al., Regulation of survival and chemoresistance by HSP90AA1 in ovarian cancer SKOV3 cells. Molecular Biology Reports, 2013. 40(1): p. 1-6.

24. Dai, H.Y., et al., Carbonic anhydrase III promotes transformation and invasion capability in hepatoma cells through FAK signaling pathway. Mol Carcinog, 2008. 47(12): p. 956-63.

25. Dai, R.P., et al., Histone 2B (H2B) expression is confined to a proper NAD+/NADH redox status. J Biol Chem, 2008. 283(40): p. 26894-901.

26. Delanote, V., J. Vandekerckhove, and J. Gettemans, Plastins: versatile modulators of actin organization in (patho)physiological cellular processes. Acta Pharmacol Sin, 2005. 26(7): p. 769-79.

27. Demali, K.A., Vinculin--a dynamic regulator of cell adhesion. Trends Biochem Sci, 2004. 29(11): p. 565-7.

28. Ding, Z., Y.H. Bae, and P. Roy, Molecular insights on context-specific role of profilin-1 in cell migration. Cell Adhesion & Migration, 2012. 6(5): p. 442-449.

29. Djinovic-Carugo, K., et al., The spectrin repeat: a structural platform for cytoskeletal protein assemblies. FEBS Letters, 2002. 513(1): p. 119-123.

30. Doe, R.P., et al., Estrogen Dosage Effects on Serum Proteins: A Longitudinal Study. The Journal of Clinical Endocrinology & Metabolism, 1967. 27(8): p. 1081-1086.

31. Dubourg, C., et al., Evaluation of ETF1/eRF1, mapping to 5q31, as a candidate myeloid tumor suppressor gene. Cancer Genetics and Cytogenetics, 2002. 134(1): p. 33-37.

32. Emori, M., et al., High expression of CD109 antigen regulates the phenotype of cancer stem-like cells/cancer-initiating cells in the novel epithelioid sarcoma cell line ESX and is related to poor prognosis of soft tissue sarcoma. PLoS One, 2013. 8(12).

33. Esteller, M., Non-coding RNAs in human disease. Nat Rev Genet, 2011. 12(12): p. 861-874.

34. Flavin, P., et al., RuvBl2 cooperates with Ets2 to transcriptionally regulate hTERT in colon cancer. FEBS Letters, 2011. 585(15): p. 2537-2544.

35. Fong, S., et al., Functional identification of distinct sets of antitumor activities mediated by the FKBP gene family. Proceedings of the National Academy of Sciences of the United States of America, 2003. 100(24): p. 14253-14258.

36. Garg, M., G. Braunstein, and H.P. Koeffler. LAMC2 as a therapeutic target for cancers. 2014.

37. Garneau, D., et al., Heterogeneous Nuclear Ribonucleoprotein F/H Proteins Modulate the Alternative Splicing of the Apoptotic Mediator Bcl-x. Journal of Biological Chemistry, 2005. 280(24): p. 22641-22650.

38. Gaude, E. and C. Frezza, Defects in mitochondrial metabolism and cancer. Cancer & Metabolism, 2014. 2: p. 10-10.

39. Ghiselli, G., SMC3 knockdown triggers genomic instability and p53-dependent apoptosis in human and zebrafish cells. Mol Cancer, 2006. 5: p. 52.

40. Gilkes, D.M., et al., Procollagen lysyl hydroxylase 2 is essential for hypoxia-induced breast cancer metastasis. Mol Cancer Res, 2013. 11(5): p. 456-66.

41. Gomes-Giacoia E, Miyake M, Goodison S, Rosser CJ. Targeting Plasminogen Activator Inhibitor-1 Inhibits Angiogenesis and Tumor Growth in a Human Cancer Xenograft Model. Molecular cancer therapeutics. 2013;12(12):2697-708.

42. Guo, C., et al., ACTB in cancer. Clinica Chimica Acta, 2013. 417: p. 39-44.

43. Gupta, D. and C.G. Lis, Pretreatment serum albumin as a predictor of cancer survival: a systematic review of the epidemiological literature. Nutr J, 2010. 9: p. 69.

44. Haase, M. and G. Fitze, HSP90AB1: Helping the good and the bad. Gene, 2015.

45. Habashy, H.O., et al., Transferrin receptor (CD71) is a marker of poor prognosis in breast cancer and can predict response to tamoxifen. Breast Cancer Research And Treatment, 2010. 119(2): p. 283-293.

46. Hamm, A., et al., Frequent expression loss of Inter-alpha-trypsin inhibitor heavy chain (ITIH) genes in multiple human solid tumors: a systematic expression analysis. BMC Cancer, 2008. 8(25): p. 1471-2407.

47. Helleday, T., et al., DNA repair pathways as targets for cancer therapy. Nat Rev Cancer, 2008. 8(3): p. 193-204.

48. Hiroshima, Y., et al., Collapsin response mediator protein 4 expression is associated with liver metastasis and poor survival in pancreatic cancer. Ann Surg Oncol, 2013. 20 Suppl 3: p. S369-78.

49. Hoeflich, K.P. and M. Ikura, Radixin: cytoskeletal adopter and signaling protein. Int J Biochem Cell Biol, 2004. 36(11): p. 2131-6.

50. Holloway, R.W., et al., Stromal Adipocyte Enhancer-binding Protein (AEBP1) Promotes Mammary Epithelial Cell Hyperplasia via Proinflammatory and Hedgehog Signaling. Journal of Biological Chemistry, 2012. 287(46): p. 39171-39181.
51. Honma, K., et al., RPN2 gene confers docetaxel resistance in breast cancer. Nat Med, 2008. 14(9): p. 939-48.
52. Hu, C.E., et al., The RNA-binding protein PCBP2 facilitates gastric carcinoma growth by targeting miR-34a. Biochem Biophys Res Commun, 2014. 448(4): p. 437-42.
53. Hu, L., et al., Biglycan enhances gastric cancer invasion by activating FAK signaling pathway. Oncotarget, 2014. 5(7): p. 1885-1896.
54. Hua, M., et al., CAP1 is overexpressed in human epithelial ovarian cancer and promotes cell proliferation. Int J Mol Med, 2015. 35(4): p. 941-9.
55. Huang, H., et al., [Establishment of drug resistant cell line of MGC-803 and analysis of differential secretome]. Beijing Da Xue Xue Bao, 2014. 46(2): p. 183-9.
56. Huang, J., et al., HMGB1 Promotes Drug Resistance in Osteosarcoma. Cancer Research, 2012. 72(1): p. 230-238.
57. Huh, Y.H., et al., Swiprosin-1 stimulates cancer invasion and metastasis by increasing the Rho family of GTPase signaling. Oncotarget, 2015. 6(15): p. 13060-13071.
58. Ji, W., et al., AIP1 Expression in Tumor Niche Suppresses Tumor Progression and Metastasis. Cancer Res, 2015. 75(17): p. 3492-504.
59. Jian, J., et al., Fibromodulin promoted in vitro and in vivo angiogenesis. Biochemical and Biophysical Research Communications, 2013. 436(3): p. 530-535.
60. Jones, N., et al., Tie receptors: new modulators of angiogenic and lymphangiogenic responses. Nature Reviews. Molecular Cell Biology, 2001. 2(4): p. 257-267.
61. Kang, J.U., et al., AMY2A: a possible tumor-suppressor gene of 1p21.1 loss in gastric carcinoma. Int J Oncol, 2010. 36(6): p. 1429-35.
62. Kanwal, R., et al., Protection against oxidative DNA damage and stress in human prostate by glutathione S-transferase P1. Molecular Carcinogenesis, 2014. 53(1): p. 8-18.
63. Katada, K., et al., Plectin promotes migration and invasion of cancer cells and is a novel prognostic marker for head and neck squamous cell carcinoma. Journal of Proteomics, 2012. 75(6): p. 1803-1815.
64. Katzenellenbogen, R.A., et al., Cytoplasmic Poly(A) Binding Proteins Regulate Telomerase Activity and Cell Growth in Human Papillomavirus Type 16 E6-Expressing Keratinocytes. Journal of Virology, 2010. 84(24): p. 12934-12944.
65. Kawabe, T., et al., Differential regulation of human RecQ family helicases in cell transformation and cell cycle. Oncogene, 2000. 19(41): p. 4764-72.
66. Kennedy, D., et al., Regulation of apoptosis by heat shock proteins. IUBMB Life, 2014. 66(5): p. 327-338.
67. Kim, H., et al., Increased CD13 Expression Reduces Reactive Oxygen Species, Promoting Survival of Liver Cancer Stem Cells via an Epithelial-Mesenchymal Transition-like Phenomenon. Annals of Surgical Oncology, 2012. 19(3): p. 539-548.

68. Kim, Y.C., R. Gonzalez-Nieves, and M.L. Cutler, Rsu1 contributes to cell adhesion and spreading in MCF10A cells via effects on P38 map kinase signaling. Cell Adh Migr, 2015. 9(3): p. 227-32.

69. Klier, F.G., et al., Amyloid ?-protein precursor is associated with extracellular matrix. Brain Research, 1990. 515(1-2): p. 336-342.

70. Kobayashi, T., et al., Activation of the ribosomal protein L13 gene in human gastrointestinal cancer. Int J Mol Med, 2006. 18(1): p. 161-70.

71. Kovac, S., G.J. Anderson, and G.S. Baldwin, Gastrins, iron homeostasis and colorectal cancer. Biochimica et Biophysica Acta (BBA) - Molecular Cell Research, 2011. 1813(5): p. 889-895.

72. Kurian, J.R., et al., Reductive detoxification of arylhydroxylamine carcinogens by human NADH cytochrome b5 reductase and cytochrome b5. Chem Res Toxicol, 2006. 19(10): p. 1366-73.

73. Lai, M.-C., et al., DDX3 Regulates Cell Growth through Translational Control of Cyclin E1. Molecular and Cellular Biology, 2010. 30(22): p. 5444-5453.

74. Langhoff, E., et al., Cathepsin D expression in pancreatic ductal adenocarcinoma (PDAC) cells increases proliferation and reduces survival of pancreatic cancer patients. Pancreatology. 15(3): p. S20.

75. Lee, I.H., et al., Ahnak functions as a tumor suppressor via modulation of TGFbeta/Smad signaling pathway. Oncogene, 2014. 33(38): p. 4675-84.

76. Lee, S.Y., et al., Microtubule-associated protein 1B light chain (MAP1B-LC1) negatively regulates the activity of tumor suppressor p53 in neuroblastoma cells. FEBS Lett, 2008. 582(19): p. 2826-32.

77. Li, J., et al., miR-27a regulates cisplatin resistance and metastasis by targeting RKIP in human lung adenocarcinoma cells. Molecular Cancer, 2014. 13: p. 193.

78. Li, L., et al., Ribonuclease inhibitor up-regulation inhibits the growth and induces apoptosis in murine melanoma cells through repression of angiogenin and ILK/PI3K/AKT signaling pathway. Biochimie, 2014. 103: p. 89-100.

79. Li, M., et al., Alpha-fetoprotein receptor as an early indicator of HBx-driven hepatocarcinogenesis and its applications in tracing cancer cell metastasis. Cancer Letters, 2013. 330(2): p. 170-180.

80. Li, X., et al., Characterization of adult alpha- and beta-globin elevated by hydrogen peroxide in cervical cancer cells that play a cytoprotective role against oxidative insults. PLoS One, 2013. 8(1): p. 17.

81. Li, X.-H., et al., Knockdown of creatine kinase B inhibits ovarian cancer progression by decreasing glycolysis. The International Journal of Biochemistry & Cell Biology, 2013. 45(5): p. 979-986.

82. Li, Z., et al., The VHL protein recruits a novel KRAB-A domain protein to repress HIF-1? transcriptional activity. The EMBO Journal, 2003. 22(8): p. 1857-1867.

83. Lindner, I., et al., ?2-Macroglobulin Inhibits the Malignant Properties of Astrocytoma Cells by Impeding ?-Catenin Signaling. Cancer Research, 2010. 70(1): p. 277-287.

84. Liu, H., et al., RKIP promotes cisplatin-induced gastric cancer cell death through NF-kappaB/Snail pathway. Tumour Biol, 2015. 36(3): p. 1445-53.

85. Liu, T.-A., et al., 14-3-3? Overexpression Contributes to Epithelial-Mesenchymal Transition of Hepatocellular Carcinoma. PLoS ONE, 2013. 8(3): p. e57968.

86. Liu, X.F., et al., A primate-specific POTE-actin fusion protein plays a role in apoptosis. Apoptosis, 2009. 14(10): p. 1237-44.

87. Liu, Z., et al., PDZ and LIM domain protein 1(PDLIM1)/CLP36 promotes breast cancer cell migration, invasion and metastasis through interaction with alpha-actinin. Oncogene, 2015. 34(10): p. 1300-11.

88. Lo, Y.-W., et al., Mitochondrial proteomics with siRNA knockdown to reveal ACAT1 and MDH2 in the development of doxorubicin-resistant uterine cancer. Journal of Cellular and Molecular Medicine, 2015. 19(4): p. 744-759.

89. Lokman, N.A., et al., Annexin A2 is regulated by ovarian cancer-peritoneal cell interactions and promotes metastasis. Oncotarget, 2013. 4(8): p. 1199-1211.

90. Lu, D., et al., Cytoskeletal protein 4.1G is a binding partner of the metabotropic glutamate receptor subtype 1 alpha. J Neurosci Res, 2004. 78(1): p. 49-55.

91. Lynch, D.K., et al., A Cortactin-CD2-associated protein (CD2AP) complex provides a novel link between epidermal growth factor receptor endocytosis and the actin cytoskeleton. J Biol Chem, 2003. 278(24): p. 21805-13.

92. Lyssiotis, C., et al., Pancreatic cancers depend on a non-canonical glutamine metabolism pathway. Cancer & Metabolism, 2014. 2(1): p. 1-1.

93. Lyssiotis, C.A., et al., Pancreatic cancers rely on a novel glutamine metabolism pathway to maintain redox balance. Cell Cycle, 2013. 12(13): p. 1987-1988.

94. Marti, A., et al., Actin-binding protein-280 binds the stress-activated protein kinase (SAPK) activator SEK-1 and is required for tumor necrosis factor-alpha activation of SAPK in melanoma cells. J Biol Chem, 1997. 272(5): p. 2620-8.

95. Maruyama, Y., et al., Short Hairpin RNA Library-Based Functional Screening Identified Ribosomal Protein L31 That Modulates Prostate Cancer Cell Growth via p53 Pathway. PLoS ONE, 2014. 9(10): p. e108743.

96. McCarroll, J. and M. Kavallaris, Microtubules, Drug Resistance, and Tumorigenesis, in Cytoskeleton and Human Disease, M. Kavallaris, Editor. 2012, Humana Press. p. 223-240.

97. McLennan, A.G., The Nudix hydrolase superfamily. Cellular And Molecular Life Sciences: CMLS, 2006. 63(2): p. 123-143.

98. McMahon, H.T. and E. Boucrot, Molecular mechanism and physiological functions of clathrin-mediated endocytosis. Nature Reviews. Molecular Cell Biology, 2011. 12(8): p. 517-533.

99. Melchior, A., et al., Cyclophilin B induces integrin-mediated cell adhesion by a mechanism involving CD98-dependent activation of protein kinase C-delta and p44/42 mitogen-activated protein kinases. Exp Cell Res, 2008. 314(3): p. 616-28.

100. Mellor, A.L. and D.H. Munn, Tryptophan catabolism and T-cell tolerance: immunosuppression by starvation? Immunology Today, 1999. 20(10): p. 469-473.

101. Mendez, M. G., Kojima, S.-I. & Goldman, R. D. 2010. Vimentin induces changes in cell shape, motility, and adhesion during the epithelial to mesenchymal transition. The FASEB Journal, 24, 1838-1851.

102. Mercurius, K.O. and A.O. Morla, Cell adhesion and signaling on the fibronectin 1st type III repeat; requisite roles for cell surface proteoglycans and integrins. BMC Cell Biol, 2001. 2(18): p. 20.

103. Mirando, A.C., et al., Aminoacyl-tRNA synthetase dependent angiogenesis revealed by a bioengineered macrolide inhibitor. Sci Rep, 2015. 5(13160).

104. Modelska, A., et al., The malignant phenotype in breast cancer is driven by eIF4A1-mediated changes in the translational landscape. Cell Death Dis, 2015. 22(6): p. 542.

105. Moon, J.-S., et al., Krüppel-like factor 4 (KLF4) activates the transcription of the gene for the platelet isoform of phosphofructokinase (PFKP) in breast cancer. The Journal Of Biological Chemistry, 2011. 286(27): p. 23808-23816.

106. Moore, A.E., et al., Physiological networks and disease functions of RNA-binding protein AUF1. Wiley Interdisciplinary Reviews. RNA, 2014. 5(4): p. 549-564.

107. Moseley, J.B., et al., Twinfilin is an actin-filament-severing protein and promotes rapid turnover of actin structures in vivo. J Cell Sci, 2006. 119(Pt 8): p. 1547-57.

108. Moudry, P., et al., Ubiquitin-activating enzyme UBA1 is required for cellular response to DNA damage. Cell Cycle, 2012. 11(8): p. 1573-82.

109. Nakanishi, K., et al., Localization and function in endoplasmic reticulum stress tolerance of ERdj3, a new member of Hsp40 family protein. Cell Stress & Chaperones, 2004. 9(3): p. 253-264.

110. Neradil, J. and R. Veselska, Nestin as a marker of cancer stem cells. Cancer Sci, 2015. 106(7): p. 803-11.

111. Onda, M., et al., Decreased expression of haemoglobin beta (HBB) gene in anaplastic thyroid cancer and recovery of its expression inhibits cell growth. Br J Cancer, 2005. 92(12): p. 2216-2224.

112. Palmieri F. Mitochondrial transporters of the SLC25 family and associated diseases: a review. Journal of Inherited Metabolic Disease. 2014;37(4):565-75.

113. Pandey, P., et al., Amyloid precursor-like protein 2 (APLP2) affects the actin cytoskeleton and increases pancreatic cancer growth and metastasis. Oncotarget, 2015. 6(4): p. 2064-75.

114. Pappas, C.T., et al., The Nebulin family: an actin support group. Trends in Cell Biology, 2011. 21(1): p. 29-37.

115. Paris, S., et al., Inhibition of tumor growth and metastatic spreading by overexpression of inter-alpha-trypsin inhibitor family chains. International Journal of Cancer, 2002. 97(5): p. 615-620.

116. Pio, R., D. Ajona, and J.D. Lambris, Complement inhibition in cancer therapy. Seminars in Immunology, 2013. 25(1): p. 54-64.

117. Popowicz, G.M., et al., Filamins: promiscuous organizers of the cytoskeleton. Trends Biochem Sci, 2006. 31(7): p. 411-9.

118. Pyper, S.R., et al., PRIC295, a Nuclear Receptor Coactivator, Identified from PPAR?-Interacting Cofactor Complex. PPAR Research, 2010. 2010: p. 173907.

119. Riley, L.G., et al., Mutation of the mitochondrial tyrosyl-tRNA synthetase gene, YARS2, causes myopathy, lactic acidosis, and sideroblastic anemia-MLASA syndrome. The American Journal of Human Genetics, 2010. 87(1): p. 52-59.

120. Rohde, M., et al., Members of the heat-shock protein 70 family promote cancer cell growth by distinct mechanisms. Genes & Development, 2005. 19(5): p. 570-582.

121. Sakamoto, H., et al., Glyoxalase I is involved in resistance of human leukemia cells to antitumor agent-induced apoptosis. Blood, 2000. 95(10): p. 3214-8.

122. Sakwe, A.M., et al., Annexin A6 contributes to the invasiveness of breast carcinoma cells by influencing the organization and localization of functional focal adhesions. Experimental cell research, 2011. 317(6): p. 823-837.

123. Seaman, M.N.J., et al., Endosome to Golgi Retrieval of the Vacuolar Protein Sorting Receptor, Vps10p, Requires the Function of the VPS29, VPS30, and VPS35 Gene Products. The Journal of Cell Biology, 1997. 137(1): p. 79-92.

124. Shimokawa, T., et al., Identification of TOMM34, which shows elevated expression in the majority of human colon cancers, as a novel drug target. Int J Oncol, 2006. 29(2): p. 381-6.

125. Singer, R., et al., Drosophila COP9 signalosome subunit 7 interacts with multiple genomic loci to regulate development. Nucleic Acids Res, 2014. 42(15): p. 9761-70.

126. Singh, P., C. Carraher, and J.E. Schwarzbauer, Assembly of Fibronectin Extracellular Matrix. Annual review of cell and developmental biology, 2010. 26: p. 397-419.

127. Stordal, B., et al., Resistance to Paclitaxel in a Cisplatin-Resistant Ovarian Cancer Cell Line Is Mediated by P-Glycoprotein. PLoS ONE, 2012. 7(7): p. e40717.

128. Storr, S.J., et al., Calpain-2 expression is associated with response to platinum based chemotherapy, progression-free and overall survival in ovarian cancer. Journal of Cellular and Molecular Medicine, 2012. 16(10): p. 2422-2428.

129. Sudo, H., et al., Knockdown of COPA, identified by loss-of-function screen, induces apoptosis and suppresses tumor growth in mesothelioma mouse model. Genomics, 2010. 95(4): p. 210-6.

130. Sugimachi, K., et al., Aberrant Expression of Plastin-3 Via Copy Number Gain Induces the Epithelial-Mesenchymal Transition in Circulating Colorectal Cancer Cells. Annals of Surgical Oncology, 2014. 21(11): p. 3680-3690.

131. Sun, Y., J. Long, and Y. Zhou, Angiopoietin-like 4 promotes melanoma cell invasion and survival through aldolase A. Oncol Lett, 2014. 8(1): p. 211-217.

132. Sung, S.Y., et al., Targeting L1 cell adhesion molecule expression using liposome-encapsulated siRNA suppresses prostate cancer bone metastasis and growth. Oncotarget, 2014. 5(20): p. 9911-29.

133. Tedeschi, P.M., et al., Contribution of serine, folate and glycine metabolism to the ATP, NADPH and purine requirements of cancer cells. Cell Death & Disease, 2013. 4: p. e877-e877.

134. Tennant, D. and E. Gottlieb, HIF prolyl hydroxylase-3 mediates alpha-ketoglutarate-induced apoptosis and tumor suppression. Journal of Molecular Medicine, 2010. 88(8): p. 839-849.

135. Tolosano, E. and F. Altruda, Hemopexin: structure, function, and regulation. DNA And Cell Biology, 2002. 21(4): p. 297-306.

136. Tondeleir, D., J. Vandekerckhove, and C. Ampe, Actin and Actin Filaments, in eLS. 2011, John Wiley & Sons, Ltd.

137. Tseng, H.Y., et al., The melanoma-associated antigen MAGE-D2 suppresses TRAIL receptor 2 and protects against TRAIL-induced apoptosis in human melanoma cells. Carcinogenesis, 2012. 33(10): p. 1871-81.

138. van der Meijden, C.M., et al., Selective expression of specific histone H4 genes reflects distinctions in transcription factor interactions with divergent H4 promoter elements. Biochim Biophys Acta, 1998. 23(1): p. 82-100.

139. van der Watt, P.J., et al., The Karyopherin proteins, Crm1 and Karyopherin ?1, are overexpressed in cervical cancer and are critical for cancer cell survival and proliferation. International Journal of Cancer, 2009. 124(8): p. 1829-1840.

140. Van Slambrouck, S., et al., Reorganization of the integrin alpha2 subunit controls cell adhesion and cancer cell invasion in prostate cancer. Int J Oncol, 2009. 34(6): p. 1717-26.

141. Velusamy, T., et al., Recurrent reciprocal RNA chimera involving YPEL5 and PPP1CB in chronic lymphocytic leukemia. Proceedings of the National Academy of Sciences, 2013. 110(8): p. 3035-3040.

142. Vie, N., et al., Overexpression of phosphoserine aminotransferase PSAT1 stimulates cell growth and increases chemoresistance of colon cancer cells. Mol Cancer, 2008. 7(14): p. 1476-4598.

143. Wang, S. and M.D. Basson, Integrin-linked kinase: a multi-functional regulator modulating extracellular pressure-stimulated cancer cell adhesion through focal adhesion kinase and AKT. Cell Oncol, 2009. 31(4): p. 273-89.

144. Wang, W., et al., Mechanisms of acquired chemoresistance to 5-fluorouracil and tomudex: thymidylate synthase dependent and independent networks. Cancer Chemotherapy and Pharmacology, 2007. 59(6): p. 839-845.

145. Wang, X., et al., Identification of triosephosphate isomerase as an anti-drug resistance agent in human gastric cancer cells using functional proteomic analysis. J Cancer Res Clin Oncol, 2008. 134(9): p. 995-1003.

146. Wang, Z.Q., et al., BCAT1 expression associates with ovarian cancer progression: possible implications in altered disease metabolism. Oncotarget, 2015. 10: p. 10.

147. Welsh, S.J., et al., The redox protein thioredoxin-1 (Trx-1) increases hypoxia-inducible factor 1alpha protein expression: Trx-1 overexpression results in increased vascular endothelial growth factor production and enhanced tumor angiogenesis. Cancer Res, 2002. 62(17): p. 5089-95.

148. Willmann, L., et al., Exometabolom analysis of breast cancer cell lines: Metabolic signature. Scientific Reports, 2015. 5: p. 13374.

149. Wu, F.-H., et al., Extracellular HSPA1A promotes the growth of hepatocarcinoma by augmenting tumor cell proliferation and apoptosis-resistance. Cancer Letters, 2012. 317(2): p. 157-164.

150. Wu, W., et al., Catechol-O-methyltransferase, a new target for pancreatic cancer therapy. Cancer Sci, 2015. 106(5): p. 576-83.

151. Wu, X., et al., Molecular mechanisms of fatty acid synthase (FASN)-mediated resistance to anti-cancer treatments. Advances in Biological Regulation, 2014. 54: p. 214-221.

152. Xu, J., S. Lamouille, and R. Derynck, TGF-beta-induced epithelial to mesenchymal transition. Cell Res, 2009. 19(2): p. 156-72.

153. Xu, Y., et al., Cell type-restricted activity of hnRNPM promotes breast cancer metastasis via regulating alternative splicing. Genes Dev, 2014. 28(11): p. 1191-203.

154. Yang, Z., et al., Adenine Nucleotide (ADP/ATP) Translocase 3 Participates in the Tumor Necrosis Factor-induced Apoptosis of MCF-7 Cells. Molecular Biology of the Cell, 2007. 18(11): p. 4681-4689.

155. Young, J.C., et al., The nuclear import factor importin ?4 can protect against oxidative stress. Biochimica et Biophysica Acta (BBA) - Molecular Cell Research, 2013. 1833(10): p. 2348-2356.

156. Zamanian-Daryoush, M., et al., The Cardioprotective Protein Apolipoprotein A1 Promotes Potent Anti-tumorigenic Effects. Journal of Biological Chemistry, 2013. 288(29): p. 21237-21252.

157. Zhang, X., et al., Kinectin-mediated endoplasmic reticulum dynamics supports focal adhesion growth in the cellular lamella. Journal of Cell Science, 2010. 123(22): p. 3901-3912.

158. Zhang, Y., et al., GNAI3 inhibits tumor cell migration and invasion and is post-transcriptionally regulated by miR-222 in hepatocellular carcinoma. Cancer Letters, 2015. 356(2, Part B): p. 978-984.

159. Zhao, C., et al., Circulating haptoglobin is an independent prognostic factor in the sera of patients with epithelial ovarian cancer. Neoplasia (New York, N.Y.), 2007. 9(1): p. 1-7.

160. Zhi, X., et al., RNAi-mediated CD73 suppression induces apoptosis and cell-cycle arrest in human breast cancer cells. Cancer Science, 2010. 101(12): p. 2561-2569.

161. Zhou, J., et al., Downregulation of LRPPRC induces apoptosis in prostate cancer cells through the mitochondria-mediated pathway. Cancer Biother Radiopharm, 2014. 29(9): p. 345-50.

162. Zimmermann, R., L. Müller, and B. Wullich, Protein transport into the endoplasmic reticulum: mechanisms and pathologies. Trends in Molecular Medicine, 2006. 12(12): p. 567-573.
